# A common allosteric mechanism regulates homeostatic inactivation of auxin and gibberellin

**DOI:** 10.1038/s41467-020-16068-0

**Published:** 2020-05-01

**Authors:** Sayaka Takehara, Shun Sakuraba, Bunzo Mikami, Hideki Yoshida, Hisako Yoshimura, Aya Itoh, Masaki Endo, Nobuhisa Watanabe, Takayuki Nagae, Makoto Matsuoka, Miyako Ueguchi-Tanaka

**Affiliations:** 1 Bioscience and Biotechnology Centre, Nagoya University, Nagoya, 464-8601 Japan; 2 Molecular Modelling and Simulation Group, National Institutes for Quantum and Radiological Science and Technology, Kizugawa, Japan; 3 Division of Applied Life Sciences, The Graduate School of Agriculture, Kyoto University, Uji, 611-0011 Japan; 4 Plant Genome Engineering Research Unit, Institute of Agrobiological Sciences, National Agriculture and Food Research Organization, Tsukuba, 305-8602 Japan; 5 Synchrotron Radiation Research Centre, Nagoya University, Nagoya, 464-8601 Japan

**Keywords:** Hormones, Plant hormones, Plant molecular biology, X-ray crystallography

## Abstract

Allosteric regulation is protein activation by effector binding at a site other than the active site. Here, we show via X-ray structural analysis of gibberellin 2-oxidase 3 (GA2ox3), and auxin dioxygenase (DAO), that such a mechanism maintains hormonal homeostasis in plants. Both enzymes form multimers by interacting via GA_4_ and indole-3-acetic acid (IAA) at their binding interface. Via further functional analyses we reveal that multimerization of these enzymes gradually proceeds with increasing GA_4_ and IAA concentrations; multimerized enzymes have higher specific activities than monomer forms, a system that should favour the maintenance of homeostasis for these phytohormones. Molecular dynamic analysis suggests a possible mechanism underlying increased GA2ox3 activity by multimerization—GA_4_ in the interface of oligomerized GA2ox3s may be able to enter the active site with a low energy barrier. In summary, homeostatic systems for maintaining GA and IAA levels, based on a common allosteric mechanism, appear to have developed independently.

## Abstract (web summary)

Enzymatic inactivation of gibberellins and auxin, via GA2ox3 and DAO respectively, contributes to hormone homeostasis in plants. Here Takehara et al. show that both enzymes multimerize in a substrate-concentration-dependent manner, and that this multimerization leads to increased enzyme activity.

## Introduction

“To grow or not to grow” is a very important decision for plant survival. Plants require suitable conditions for growth, in the absence of which growth needs to cease. Thus, they have developed effective systems to control the amount of the growth hormones, gibberellin (GA) and auxin (IAA), by regulating the levels of the corresponding synthetic/inactivation enzymes. In fact, the transcription of GA- and IAA-biosynthesis or breakdown-related genes is strictly regulated by the level of active GA and IAA as a negative feedback or as a positive feedforward mechanism^1–4^.

In 1961, Monod and co-workers^5–9^ proposed the “allosteric” theory to explain feedback inhibition of bacterial enzymes. The word is composed of two Greek roots that reflect the difference (allo-) and specificity (stereo-) of the two binding sites. The concept of this theory is based on the fact that the activity of proteins is regulated by binding an effector molecule at a site other than the active site, whereby a conformational change occurs, which in turn causes hyperactivation or inactivation. Such allosteric regulation is a natural example of control feedback loops to maintain homeostasis at the post-translational level. However, to our knowledge, there have been no reports to date on the maintenance of homeostasis of phytohormone levels through allosteric regulation, although maintaining phytohormone homeostasis at the post-translational level is surely as important for plant survival as it has been shown to be for the regulation of enzyme activity in bacteria^10^.

Here, we studied the post-translational regulation of GA and IAA inactivation enzymes from the viewpoint of their allostery and the homeostasis of GA and IAA levels. We performed X-ray structural and functional analyses of GA and IAA inactivation enzymes in rice, GA 2-oxidase 3 (OsGA2ox3) and dioxygenase, for auxin oxidation (OsDAO), respectively. The results revealed that a monomer–multimer switching occurs based on a substrate level, and that the resulting conformational change enhances the activities of these enzymes to maintain phytohormone homeostasis, in full agreement with the allosteric theory by Monod and co-workers^5–9^. Finally, we also discuss the establishment of homeostatic regulation systems for the two different plant growth hormones from the evolutionary perspective.

## Results

### X-ray structural analysis of OsGA2ox3 complexed with GA_4_

**Fig. 1:**
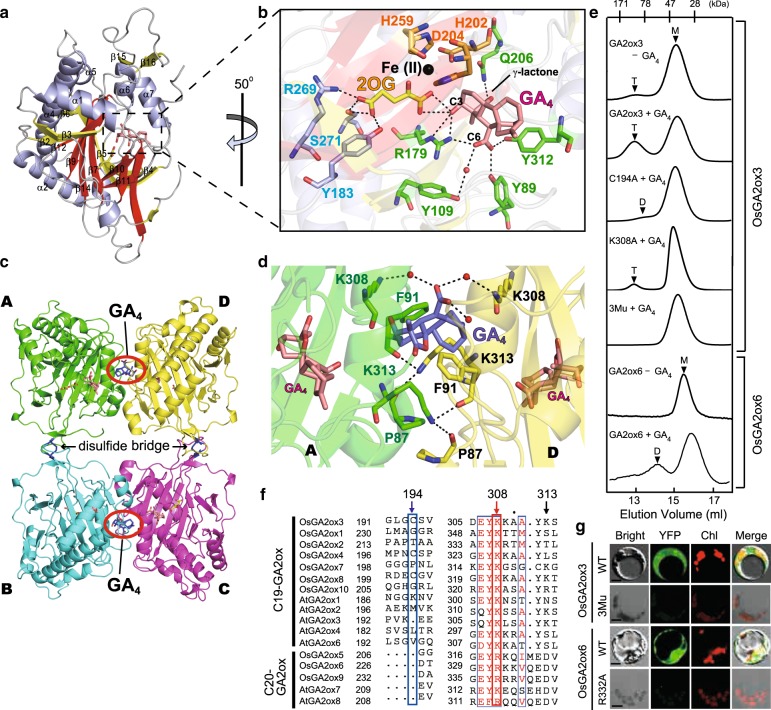
OsGA2ox3 forms GA-mediated tetramer structure. **a** Crystal structure of OsGA2ox3. The α-helices are shown in blue, the common core fold is shown in red and none core β-strands are in yellow. **b** Close-up view of the active site. Important active site residues 2OG and GA_4_ are shown in yellow and pink with stick form, respectively. Fe(II) is colored in black. The amino acids interacting with 2OG (R269, S271, and Y183 shown in blue characters), with Fe(II) (H202, D204, and H259 shown in orange characters) and with GA_4_ (Q206, Y312, Y89, Y109, and R179 shown in green characters). **c** Overall structure of OsGA2ox3 tetramer composed of four subunits A–D (see details in the text). **d** Close-up view of the interface containing GA_4_ between subunit A and D. The GA_4_ in the interface and active site of subunit A and D are shown in blue and pink, respectively. Amino acids involved in interaction with the interface GA_4_ are highlighted, whereas the hydrogen bonds mediated by two water molecules (red spheres) are also indicated by dashed lines. **e** The gel filtration profile of WT-OsGA2ox3, WT-OsGA2ox6, and their mutation derivatives with or without GA_4_. 3Mu is OsGA2ox3 carrying C194A, K308A, and K313A. T, D, and M indicate the expected elution positions for tetramers, dimers, and monomers of OsGA2oxs. **f** Alignment of amino acids involved in multimerization of GA2oxs from rice and Arabidopsis, 194th, 308th, and 313th. The residue at the position of 308 is always shared by K or R. **g** BiFC analysis of multimer formation of WT-OsGA2ox3, WT-OsGA2ox6, and their mutation derivatives. YFP yellow fluorescent protein, Chl autofluorescence of chloroplasts. Scale bar = 10 μm.

**Fig. 2:**
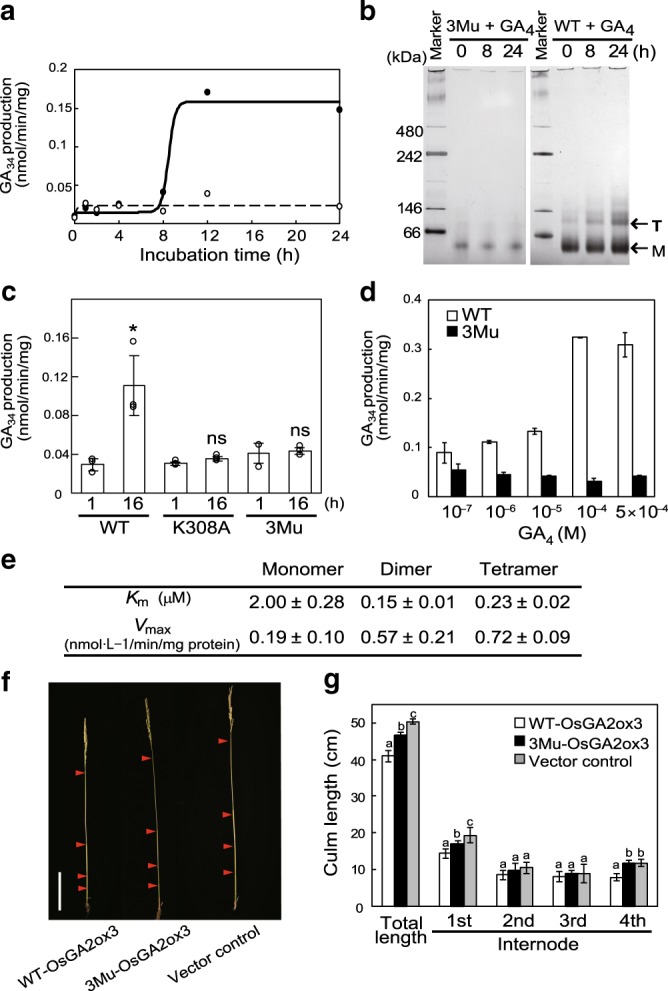
Multimerization of OsGA2ox3 enhances its activity in vitro and in planta. **a** Time course of OsGA2ox3 activation by GA_4_. The activity of WT- and 3Mu-OsGA2ox3 is shown by closed and open circles, respectively. **b** Assessment of OsGA2ox3 multimerization by blue-native PAGE. WT- and 3Mu-OsGA2ox3 are indicated with GA_4_ for 0, 8, and 24 h. Monomer (M) and tetramer (T) are indicated. **c** Enzyme analyses of OsGA2ox3, 3Mu, and K308A mutant (*n* = 3 biologically independent samples; central values and error bars represent means ± s.d.). The differences within the same category are indicated. *P < 0.05; NS not significant (*P* > 0.05); two-tailed paired *t*-tests. **d** Dose response of OsGA2ox3 and 3Mu activation by GA_4_ (*n* = 3 biologically independent samples; central values and error bars represent means ± s.d.). OsGA2ox3 (white bars) and 3Mu (black bars) were incubated for 12 h at various GA_4_ concentrations. **e** *K*_m_ and *V*_max_ of OsGA2ox3 with different multimerization forms (*n* = 3 biologically independent samples). **f** Representative internode elongation pattern shown in **g**. Red arrowheads indicate the position of nodes. Scale bar = 10 cm. **g** Complementation of the culm elongation in *ga2ox3* knockout plants by overexpression of WT- and 3Mu-*OsGA2ox3* cDNA (*n* = 6 biologically independent samples; central values and error bars represent means ± s.d.). Different letters denote significant differences (*P* < 0.05) based on the Tukey–Kramer test.

GA 2-oxidase 3 (GA2ox3) is a member of the 2-oxoglutarate (2OG)- and Fe (II)-dependent dioxygenase (2ODD) enzyme family that requires 2OG and molecular oxygen as co-substrates, and ferrous iron Fe(II) as a co-factor, to inactivate biologically active GA_4_^11,12^. Rice has 10 different *Oryza sativa* GA2oxs (OsGA2oxs) (Supplementary Fig. 1). Here, we used OsGA2ox3 for studying the molecular mechanism of homeostatic regulation because OsGA2ox3 predominantly functions in rice seedlings^13^. We crystallized the enzyme with the co-substrates, 2OG and GA_4_ in the absence Fe(II) to prevent its enzymatic action and determined its structure via X-ray crystallography at a resolution of 2.15 Å (Supplementary Table 1). The crystallized protein consisted of a homotetramer (see below), whose each subunits with a common core fold in 2ODD enzyme family (red in Fig. 1a). The overall molecular structure of the OsGA2ox3 subunit was essentially the same as that for other 2ODD enzymes reported^14,15^ (Supplementary Fig. 2a), including the 2OG-binding active site (yellow in Fig. 1b, Supplementary Fig. 2b). In fact, the amino acids interacting with 2OG (R269, S271, and Y183, shown in blue characters in Fig. 1b) were located in the same manner as in other 2ODD enzymes (Supplementary Figs. 2b and 3). As the location of amino acid residues in OsGA2ox3 interacting with Fe(II) are the same as that reported for other 2ODD enzymes, and Fe(II) was absent from crystalized OsGA2ox3, we located Fe(II) at the same position (Fig. 1b, Supplementary Fig. 2b). While GA_4_ interacted with non-conserved amino acids, such as Q206, Y312, Y89, Y109, and R179, among 2ODD enzymes (green characters in Fig. 2b and green closed triangle in Supplementary Fig. 3). Additionally, GA_4_ also interacted with 2OG located at the bottom of the active-site cavity (Fig. 2b, Supplementary Fig. 4a). Interestingly, the opening width of this cavity was too small for the substrate (GA_4_) or for the product (GA_34_) to go in or out (Supplementary Fig. 4b), indicating that there might be a specific passing mechanism involved in controlling transit in either direction (see below).

### OsGA2ox3 forms a tetramer structure

The crystalized OsGA2ox3 formed a homotetramer (A–D in Fig. 1c) by disulfide bridges (C194–C194) and hydrogen bonds bridged by the interface GA_4_s in a face-to-face manner (red circles in Fig. 1c). Figure 1d shows a close-up view around the interface GA_4_ lying between two subunits, A and D. For their interaction via GA_4_, K308s in both subunit bound to the C-6 carboxyl group of interface GA_4_ via water molecules. K313 in subunit A directly interacted with P87 and F91 in subunit D by a hydrogen bond, and vice versa.

We purified OsGA2ox3 in the absence of GA_4_ in an attempt to explain the need for GA_4_ to form the tetramer. A dominant peak of the monomer (M) was observed at a reasonable size with a very weak peak of higher molecular weight (T) in the absence of GA_4_ in a gel filtration profile (GA2ox3−GA_4_ in Fig. 1e and Supplementary Fig. 5). In contrast, peak T corresponding to the tetramer appeared in the presence of GA_4_ (GA2ox3 + GA_4_), confirming that tetramerization was induced by the presence of GA_4_. We replaced the amino acids involved in multimerization with Ala and examined its multimerization ability. Single replacement of K308 (GA dependent) or C194 (disulfide bridge), with Ala, drastically impeded tetramer formation (C194A + GA_4_ and K308A + GA_4_). Furthermore, the triple mutant carrying C194A, K308A and K313A (3Mu) did not show a tetramer peak (3Mu + GA_4_).

There are two types of GA2oxs, C19- and C20-GA2ox, which specifically act on the C19- and C20-type GAs as substrates^16^. Further, OsGA2ox3 is a member of C19-type (Supplementary Fig. 1). As GA2oxs, including C19- and C20-types, do not always contain Cys for disulfide bridge at the corresponding position (Fig. 1f, blue box), we also examined multimerization of another C20-type of GA2ox, OsGA2ox6, in the absence of Cys. The presence of GA_4_ induced a new peak of its dimer form (compare GA2ox6 ± GA_4_ in Fig. 1e), demonstrating that the Cys disulfide bridge is not required for GA-dependent multimerization. Finally, we tested multimerization in planta by BiFC analysis. Co-incubation of the wild-type (WT) OsGA2ox3s fused with N- or C-YFP in rice protoplasts showed a clear signal in the cytosol (Fig. 1g), whereas 3Mu did not (Fig. 1g). As before, a single mutation of OsGA2ox6, R332A (Fig. 1f, red box), caused failure in dimerization (Fig. 1g), thus confirming that the presence of Lys or Arg corresponding to the position at K308 on OsGA2ox3 is essential for GA-dependent multimerization.

### Multimerization stimulates GA2oxs enzymatic activity

To next address the relationship between multimerization and enzymatic activity, we examined the time course of enzymatic activity upon incubation with GA_4_. We found that a significant level of activation occurred more than 8 h after incubation with GA_4_ (Fig. 2a), whereas tetramerization was observed to some extent at 8 h and thereafter (Fig. 2b). In contrast, a single mutation, such as K308A or 3Mu, led to no activation by GA_4_ (Fig. 2a–c). Additionally, we examined the dose response to GA_4_, and found that the activation started below 10^−6^ M of GA_4_ and saturated at near 10^−4^ M, while no activation of 3Mu occurred at all (Fig. 2d).

We further performed the kinetic study of the various forms of OsGA2ox3 separated by gel filtration (Fig. 2e, Supplementary Fig.6). *K*_m_ for GA_4_ was almost 10-fold lower for the tetramer and dimer than that for the monomers, while, consistently, *V*_max_ of these multimers was more than threefold higher than that corresponding to the monomers. These findings clearly demonstrate that multimerization enhanced OsGA2ox3 activity.

Next, we studied the biological significance of the multimerization of GA2ox in planta. We generated overexpressors (*GA2OX3-OV*) driven by the actin promoter and knockout plants (*ga2ox3*) by a CRISPR/Cas9 system. *GA2OX3-OV* showed more severe dwarfism at higher levels of OsGA2ox3 expression (Supplementary Fig. 7a, b), whereas *ga2ox3* formed elongated internodes and leaves (Supplementary Fig. 7c–g), indicating that a difference in OsGA2ox3 activity reflected on the morphology of the rice plant. Furthermore, a reporter gene (GUS) controlled by the *OsGA2ox3* promoter was dominantly expressed in elongating internodes and nodes in the culm but was faintly expressed in other tissues (Supplementary Fig. 7h–k), thus suggesting that internode elongation might be the most suitable system for precise evaluation of changes in enzymatic activity in planta. Next, we examined the effect of WT-OsGA2ox3 cDNA or 3Mu cDNA driven under its own promoter on internode elongation in *ga2ox3* plants (Fig. 2f, g). At the heading stage, plants carrying WT-cDNA were the shortest in height, with shorter internodes; in contrast, plants carrying 3Mu cDNA showed an intermediate phenotype relative to control plants. This clearly demonstrated that multimerization of OsGA2ox3 changed its enzymatic function in plants growing under natural conditions (see Discussion).

### Activation mechanism of OsGA2ox by multimerization

**Fig. 3:**
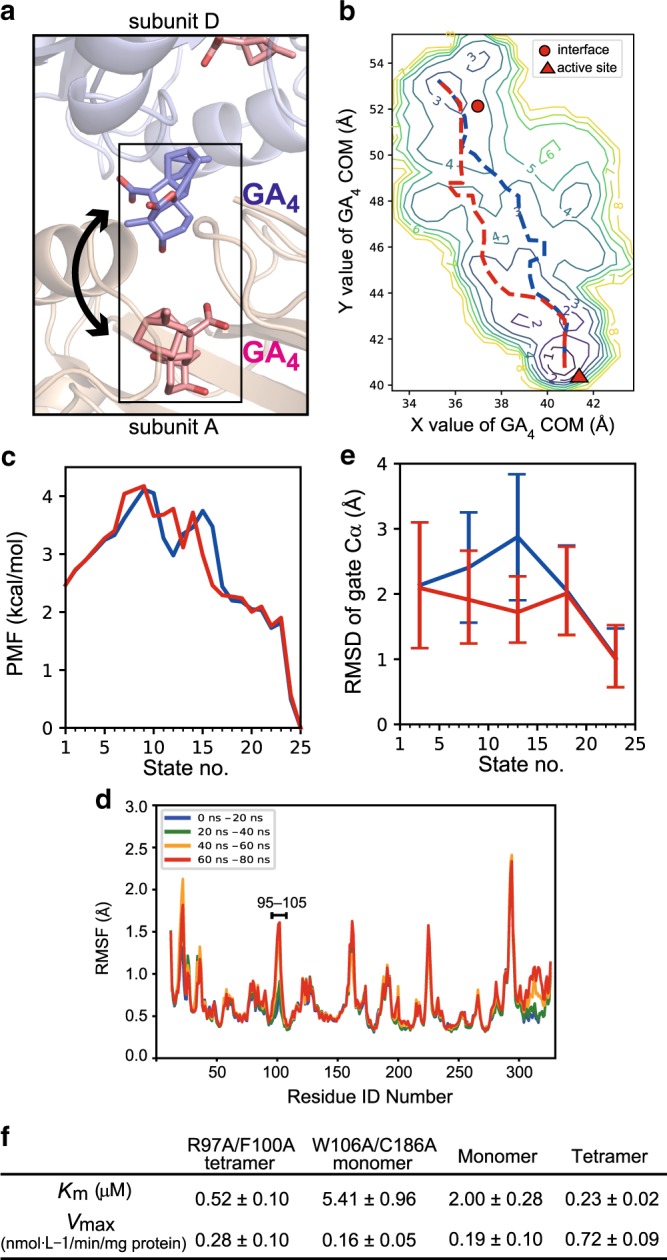
Free-energy landscape of the GA_4_ loading process. **a** Schematic view of the free-energy analysis. GA_4_ was moved between two binding sites, the interface (purple) and active site (pink). **b** The free-energy landscape of the GA_4_ loading process. Numbers represent the height of the contour in kcal/mol. The horizontal and vertical axes represent the center-of-mass (COM) coordinate of GA_4_. Two GA_4_ sites found in the X-ray crystallographic analysis were plotted as a red circle and a triangle. Broken lines (magenta and orange) represent two possible pathways connecting both sites. **c** The relative stabilities were calculated as a potential of mean force (PMF) for the predicted two pathways in panel **b**. **d** The change in the fluctuation of Cα atoms upon the GA_4_ exiting the active site. In addition to the alpha helices around K308, residues in the gate were destabilized when GA_4_ exited the active site. **e** The root-mean-square deviation (RMSD) of the gate (near residues 96–106) during the simulation (*n* = 8 biologically independent samples; central values and error bars represent means ± s.d.). **f** Kinetic analysis of monomer, dimer and tetramer of WT and mutant OsGA2ox3 (*n* = 3 biologically independent samples).

We studied the activation mechanism of OsGA2ox3 using molecular dynamics (MD) simulation based on the tetrameric structure. Note that in the absence of comparative structures for monomers this can only generate a hypothetical mechanism for activation by multimerization. By free-energy analysis, we transferred GA_4_ from the interface to the active sites (shown in purple for interface GA_4_ and in pink for active site in Fig. 3a), and estimated its relative stability as potential mean force. Our findings agreed with X-ray crystallographic results (Fig. 3b). The free-energy landscape of GA_4_ revealed the presence of two local free-energy minima around the interface and active sites (indicated with a circle and triangle in Fig. 3b, respectively), whereas the stability of GA_4_ around the active site (State no.1 in Fig. 3c) was higher than that of the interface (State no. 25 in Fig. 3c), with a 2.5 kcal/mol difference (Fig. 3c). Our results also revealed two possible pathways from the interface to the active sites (red and blue lines in Fig. 3b, c), with a nearly identical energy barrier of 1.7 kcal/mol, as estimated by the difference between peak height (4.2 kcal/mol) and the base line (2.5 kcal/mol) (Fig. 3c). These energy barriers are only slightly higher than thermal fluctuation (0.6 kcal/mol at 25 °C), indicating that such barriers can readily be surpassed without assistance from any external force. Consequently, the interface GA_4_ could be readily loaded into the active site. We predicted two possible transition states for these different pathways (Supplementary Fig. 8a) and identified key interactions to stabilize the intermediate states upon loading of GA_4_ (Supplementary Fig. 8b, c). In the red and blue pathways, K308 in subunits A and D, respectively, formed a salt bridge with the C-6 carboxyl group of GA_4_; however, an additional hydrogen bond between K308 in subunit A and γ-lactone of GA_4_ occurred only in the blue pathway (Supplementary Fig. 8c). We predict that K308 is important for moving GA_4_ from the interface to the active site. Because the opening width of the OsGA2ox3 active site is rather small (Supplementary Fig. 4b), GA_4_ of the active site likely enters and exits by dynamic fluctuations near the entrance. Therefore, we investigated the relative flexibility of various regions by simulating the exit process of GA_4_ from the active site. In addition to the structural change around K308, the root-mean-square fluctuation (RMSF) of Cα atoms indicated that residues 96–106, composed of two β-strands and a loop connecting the strands (Supplementary Fig. 9a–c), were markedly destabilized as GA exited the active site (Fig. 3d, Supplementary Fig. 9d). Next, we determined the structural dynamics of this region in the blue and red pathways over the course of GA_4_ movement by calculating the root-mean-square deviation (RMSD) (Fig. 3e). In both pathways, the region was opened upon loading GA_4_ into the active center (gate open, Supplementary Fig. 9e), and was closed when the ligand was fully loaded (gate close, Supplementary Fig. 9e); we thus termed this loop “gate”. This analysis, together with the tetramer structure, suggested that two sets of paired amino acids (R97/F100 and W106/C186) are involved in the stabilization process of the opened gate through interacting with subunits A and D as the hinge of the gate (Supplementary Fig. 9a–c). We exchanged these residues with Ala and examined the effect on the enzymatic properties (Fig. 3f). The R97A/F100A-tetramer affected both the *K*_m_ and the *V*_max_ values, whereas the W106A/C186A monomer affected only the *K*_m_. Along with MD and structural analyses, a potential activation mechanism of OsGA2ox3 by multimerization is summarized in Supplementary Fig. 9e. Together, these findings suggest the importance of the gate region.

### IAA-mediated multimerization of OsDAO triggers its activity

**Fig. 4:**
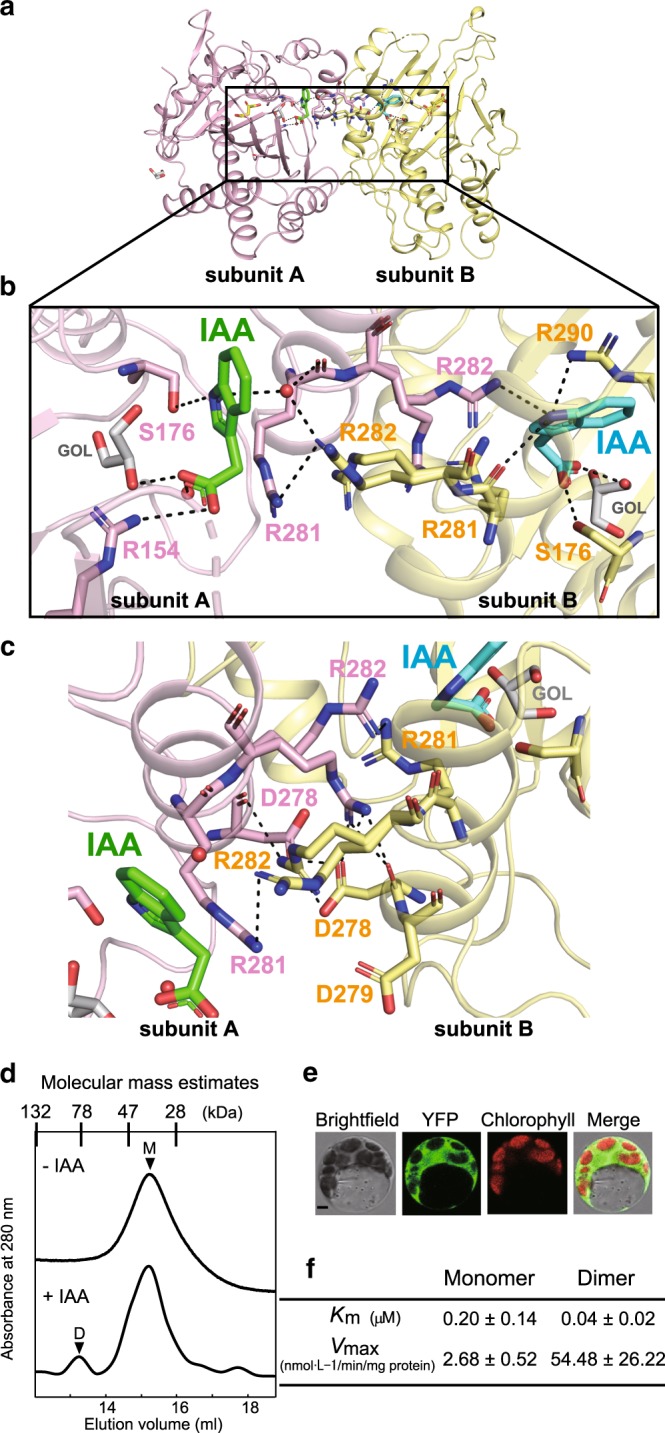
OsDAO forms an IAA-mediated dimer structure to trigger its enzyme activity. **a** Overall structure of the dimer form of OsDAO. **b**, **c** Detailed interactions at the dimer–dimer interface of the OsDAO bound to IAA (green and cyan stick forms), the residues surrounding the interface are represented by a stick model, shown as pink and yellow sticks. The hydrogen bonds mediated by water molecule (red spheres) are also indicated by dashed lines. **d** A typical elution profile of OsDAO sample under the presence of IAA condition in gel filtration. The proposed conformation of each eluted peak is indicated by M (monomer) and D (dimer). **e** BiFC analysis of dimeric interaction of DAO. Scale bar = 5 μm. **f** Kinetic analysis of monomer and dimer of OsDAO. Comparison of *K*_m_ and *V*_max_ calculated by the corresponding Lineweaver–Burk plots (*n* = 3 biologically independent samples).

Recent studies have revealed that DAO controls IAA concentration, which is important for modulating auxin homeostasis, since it is dependent on catabolizing IAA. Interestingly, all DAOs listed in Supplementary Fig. 10 share Arg or Lys at residue 308 in OsGA2ox3, which is essential for GA_4_-dependent oligomerization and hyperactivation, as previously mentioned. Thus, we hypothesized that the activity of DAO might also be regulated through multimerization via IAA. We elucidated the crystal structure of OsDAO complexed with its substrate, IAA, at a 2.0-Å resolution (Supplementary Table 1). As expected, OsDAO had a stereotype structure of 2ODD (Fig. 4a), and formed a dimer containing two IAA molecules at the interface region (Fig. 4b). An IAA contacting subunit A (green stick) interacted with R154 and S176 of subunit A and R282 of subunit B via a water molecule, while the other IAA on subunit B (cyan stick) interacted with R281, R290, and S176 of subunit B and R282 of subunit A (Fig. 4b). These subunits directly interacted with each other through hydrogen bonds at residues D278, D279, R281, and R282 (Fig. 4c).

The elution profile of gel filtration showed that dimer formation of OsDAO occurred in the presence of IAA but not in its absence (Fig. 4d). OsDAO multimerization in planta was also confirmed by BiFC analysis (Fig. 4e). *K*_m_ for IAA of the dimer was nearly fivefold lower than that of the monomer, while consistently, *V*_max_ of the dimer was 22-fold higher than that of the monomer (Fig. 4f, Supplementary Fig. 11). These observations demonstrated that the activity of an auxin-catabolizing enzyme, DAO, is regulated by the same mechanism that regulates GA2ox; namely, through substrate-mediated multimerization.

### Phylogenetic analysis of 2ODD enzymes

**Fig. 5:**
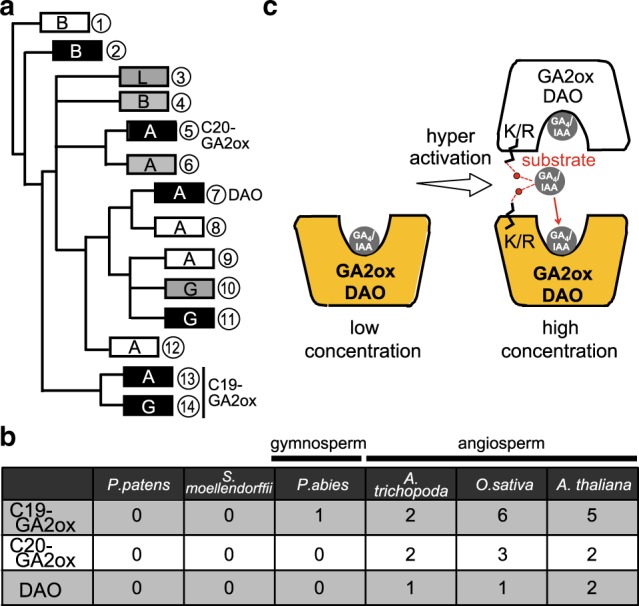
Activation model of OsGA2oxs and OsDAO and phylogenetic analysis. **a** Schematic phylogenetic tree of C19- and C20-type GA2oxs and DAOs, including some 2ODD enzymes bearing close resemblance to these enzymes. Each box corresponds to each clade with the same number presented in Supplementary Fig. 10. Clades containing only 2ODD enzymes with R/K at the 308th position of OsGA2ox3 or not are shown in black or white, whereas clades including both types are shown in gray. Bryophytes, lycophytes, gymnosperms, and angiosperms are shown as B, L, G, and A characters, respectively. **b** The number of gene copies of C19- and C20-type GA2ox and DAO in *P. patens* (Bryophytes), *S. moellendorffii* (Lycophytes), *P. abies* (Gymnosperms), *A. trichopoda* (the most primitive lineage of Angiosperms), *O. sativa* (monocot), and *A. thaliana* (dicot). **c** Under low substrate concentration (GA_4_ or IAA) (left), GA2ox or DAO functions as a monomer with low enzyme activity. At high substrate concentration (right), the interface GA_4_ or IAA causes the formation of multimers by bridging two enzyme molecules, resulting in hyperactivation.

Phylogenetic analysis of 2ODD enzymes from angiosperms (rice, *Arabidopsis thaliana*, and *Amborella trichopoda*), gymnosperms (*Picea abies*), Lycophytes (*Selaginella moellendorffii*), and Bryophytes (*Physcomitrella patens*) revealed that C19 and C20-type GA2oxs and DAO are classified into independent clades (Fig. 5a, Supplementary Fig. 10), indicating that these enzymes evolved independently. Phylogeny also suggested that the emergence of the C19-type of GA2ox might have occurred at the time when seed plants first appeared, since *P. abies* contains its homolog but *S. moellendorffii* and *P. patens* do not (Fig. 5b, Supplementary Fig. 10). In contrast, the C20-type of GA2ox and DAO may have evolved with angiosperms, because there is no existing homolog within the genome of gymnosperms, although certain homologs have been reported in *A. trichopoda*, the most primitive lineage in the angiosperm clade (Supplementary Fig. 10). Thus, plants seem to have independently developed essentially identical systems at three distinct time points over the course of evolution to regulate levels of GA_4_ and IAA (Fig. 5b).

## Discussion

Here, we determined an X-ray crystal structure and performed functional analyses of two rice 2ODD family enzymes, OsGA2ox3 and OsDAO, which catabolize plant growth hormones GA_4_ and IAA, respectively. Both enzymes formed tetramers for OsGA2ox3, and dimers for OsDAO with the aid of their substrates, thereby enhancing their activity, as schematically summarized in Fig. 5c. Synchronous structural changes and activity enhancement are typical of allosteric-regulation events, as shown in the model proposed by Monod et al.^5–9^. Based on this model, triggering of OsGA2ox3 and OsDAO activities can be explained as follows: at a low concentration of GA_4_ or IAA, each enzyme is present as a monomer or protomer (the specific term proposed by Monod) and displays steady-state activity^8^. With increasing substrate concentration, the enzyme gradually forms a multimer with the aid of the substrate itself (effecter), thereby resulting in an increase in enzymatic activity and in the active catabolism of GA or IAA to maintain hormonal homeostasis. However, further studies are needed to investigate how multimerization of OsGA2ox3 leads to increase enzyme activity because equivalent simulations on the monomer of OsGA2ox3 have not been performed and thus our proposed mechanism remains hypothetical.

Previously, Zhang et al.^17^ reported that the inactivation of IAA by AtDAO1 was more than 10,000-fold lower than inactivation by GH3.6, another IAA inactivation enzyme. They speculated that DAO1 might function spatiotemporally to terminate active IAA constitutively during development, in contrast to GH3, which responds quickly to environmental factors that increase cellular IAA levels^17^. These studies, combined with our results, provide further insight into the biological implications of the DAO-mediated auxin inactivation system. Indeed, our results showed that OsDAO activity (maximum reaction rate for oxidation of IAA at 54.48 nmol/min/mg protein) was a quarter of that reported for GH3.6 (maximum reaction rate for conjugation of Asp to IAA at 244 nmol/min/mg protein^18^), under high concentrations of IAA, to induce DAO-dimer formation. This result confirms the importance of DAO for auxin catabolism relative to GH3, and clarifies differences between these two enzymes in terms of IAA inactivation. *GH3* genes, which mainly work as an emergency-response system against environmental changes experienced by plants^19–22^, are among the fastest to respond to exogenous auxin and environmental stimuli, with transcript levels increasing by 10-fold within 1 h. In contrast, the DAO inactivation system may primarily be involved in intrinsic biological events/processes, and may modulate activity through an intramolecular mechanism according to substrate level. Effective dual systems are most likely important for plants exposed to diverse environmental conditions under continuous fluctuation. Interestingly, such a dual system was not observed in the case of the GA_4_ catabolic mechanism, suggesting that rapid inactivation may not be essential for GA-regulated biological events.

Phylogenetic analysis of 2ODD enzymes revealed that plants seem to have independently developed essentially identical systems at three separate time points over the course of evolution to regulate the level of GA_4_ and IAA (Fig. 5b). Such independent development of the homeostatic regulation systems described, at the time of seed plant and angiosperm emergence, suggests that systems such as these could be important for plant adaptation to varying environmental conditions and to effectively control levels of growth hormones. Consequently, these elegant systems have no doubt aided plant survival and contributed to better adaptation in fluctuating and challenging environments.

## Methods

### Plasmid construction

Sequences of primers used in this study are listed in Supplementary Table 2. The PCR fragments were sequenced to confirm that no mutations were induced. To construct GST-tagged proteins, the full-length coding regions for the OsGA2ox3, OsGA2ox6, and OsDAO containing appropriate restriction sites were cloned into pGEX6P1 (GE Healthcare) using primer sets (Supplementary Table 2, No. 1–6). The OsGA2ox3 expression plasmid, in which Cys194, Lys308, Lys313, Trp106, Cys187, Fhe100, and Arg97 residues of OsGA2ox3 were replaced with Ala, was constructed with one set mutagenized primers (Supplementary Table 2, No. 7–20) corresponding to each mutation and pGEX6P1/OsGA2ox3 as template.

To generate constructs of the OsGA2ox3, OsGA2ox6, and OsDAO for the transient protein expression, the full-length cDNA without a stop codon was amplified using appropriate primers (Supplementary Table 2, No. 21–28) and subcloned into the pENTR-D/TOPO gateway entry vector using the TOPO reaction according to the supplier’s directions (Invitrogen). For constructs used in the BiFC experiment, genes from pENTR- D/TOPO clones were transferred to the N-terminal half clone, pGWnY vector and the C-terminal half clone, pGWcY vector by LR Gateway reaction to generate C- and N-terminal fusions to the two YFP fragments.

To generate transgenic plants, the Act1 promoter of the pSTARA vector (Kumiai Chemical Industry) located between the gateway cloning site and the acetolactate synthase (ALS) terminator was replaced with a 1.8 kbp genomic DNA of OsGA2ox3 promoter (pOsGA2ox3) using primers with suitable restriction enzyme sites (Supplementary Table 2, No. 29 and 30). The OsGA2ox3-WT and OsGA2ox3-3Mu cDNA was cloned by LR Gateway reaction into pOsGA2ox3-pSTARA vector. The pOsGA2ox3-pSTARA vector was used as a control for transient transformation. For the promoter-GUS assay, a 2.6 kbp genomic DNA, including the cording region (exon 1 and part of exon 2) and the 5′ promoter region of the OsGA2ox3 was amplified by the primer pair (Supplementary Table 2, No. 31 and 32) using the Nipponbare genomic DNA as the template and inserted into the *Xba*I and *Sma*I sites of the binary vector pBI/Hm2 to drive the GUS reporter gene expression. For the overexpression of OsGA2x3, the OsGA2ox3 cDNA was amplified by the primer pair (Supplementary Table 2, No. 33 and 34) and inserted into the *Xba*I and *Sma*I sites of the vector pCAMBIA 1380 to drive CaMV35S promoter. To generate constructs of the OsGA2ox3 for the knockout mutant, the vectors in this study are based on Cas9 cloning vectors (pU6gRNA)^23^. The pU6gRNA has two *Bbs*I sites between the OsU6 promoter and the gRNA scaffold sequence. The 20 nt annealed oligonucleotides (Target) (Supplementary Table 2, No. 35–38) were ligated into this restriction enzyme recognition sites of the pU6gRNA. To connect gRNA expression cassettes, the gRNA expression cassette (OsU6pro- 2pro::gRNA::polyT) was eliminated from pU6gRNA by using *Pac*I and *Asc*I, and inserted into the pZDgRNA_Cas9ver.2_HPT vectors. The all plants were genotyped using primers No. 39–42 (Supplementary Table 2) by the optimized PCR.

### Expression and purification of OsGA2ox3 and OsDAO

OsGA2ox3 and OsDAO/pGEX6P1 constructs were overexpressed in *Escherichia coli* Rosetta (DE3) pLysS (Novagen). Cells were grown at 37 °C to the mid-log phase in Terrific-Broth medium containing 100 μg/ml ampicillin, followed by IPTG induction at 18 °C for 16–18 h. The harvested cells were washed with buffer A [10 mM Na-phosphate, 150 mM NaCl, 1 mM DTT (pH 7.5)] containing Complete protease inhibitor (Merck) and 1.5 mM phenylmethylsulfonyl fluoride, and then disrupted by sonication. OsGA2ox3 and OsDAO were obtained from the supernatant fraction. The recombinant protein in the supernatant was purified with Glutathione Sepharose 4B resin (GE Healthcare) equilibrated with Buffer A. The column was washed with 30 bed volumes of buffer A, and eluted with buffer A (plus 20 mM glutathione pH 7.5). To remove the GST-tag, precision protease (GE Healthcare) was added and incubated overnight at 4 °C. The GST and tag-digested OsGA2ox3 and OsDAO were filtered through a 0.22 μm nylon filter and further purified using MONOQ column (GE Healthcare) equilibrated with buffer B [10 mM Na-phosphate, 1 mM DTT] with a NaCl gradient (0–1000 mM) in buffer B. The sample was concentrated and dialyzed with buffer C (10 mM sodium phosphate, pH 7.4) using an Amicon Ultra-4 concentrator unit (10 kDa molecular weight cut-off) (Millipore). The protein concentrations of OsGA2ox3 were measured by UV absorbance at 280 nm. The *A*^0.1%^ values at 280 nm (0.90, 0.73 for the OsGA2ox3 and OsDAO, respectively) were calculated from the amino acid composition^24^. The amount of OsDAO protein in buffer containing IAA was estimated from intensity of the band in SDS-PAGE gel using Image J. Then all protein samples were run on both a 12% wt/vol SDS gel and a 3–12% wt/vol nondenaturing gel stained and visualized by Coomassie Brilliant Blue R-250 staining. The target proteins were detected by comparison with protein standard markers, and the optimum conditions for in vitro expression were determined.

### Crystallization

Screening experiments were performed with several commercial kits. Crystallization was performed by the sitting drop vapor diffusion method at 20 °C with 1 μl protein mixed with 1 μl mother liquid solution. Droplets containing 20 mg/ml OsGA2ox3 dissolved in 5 mM Na-phosphate (pH 7.5), 10 mM 2-oxoglutarate and 2 mM GA_4_ and mother liquor were equilibrated against 50 μl of reservoir solution composed of 0.2 mM ammonium citrate (pH 5.1) and 20% (w/v) PEG3350. The OsDAO was dissolved in 5 mM Na-phosphate (pH 7.5), 10 mM 2-oxoglutarate and 2 mM IAA, and mother liquor comprised 0.1 mM sodium acetate, 0.2 mM ammonium sulfate, and 22% (w/v) PEG 4000. Crystals were flash-frozen in liquid nitrogen with an addition of 30% glycerol in the crystallization of mother liquid as cryoprotectant. X-ray diffraction data were collected on BL26B1 beamline at SPring-8. All data were processed and scaled using HKL2000 (ref. ^25^). The crystal data are summarized in Supplementary Table 1.

### Structure determination and refinement

The crystal of OsGA2ox3 was calculated by a single-wavelength anomalous dispersion (SAD) method using data collection at the peak wavelength of Au. HKL2MAP^26^ was used to run the SHELX suite of programs. Au positions were located using SHELXD, then the Au sites were located with SHELXD, and single-wavelength anomalous diffraction phases to 3.0 Å resolution were calculated with SHELXE using the data collected at the X-ray wavelength (1.0 Å) corresponding to the peak of the X-ray fluorescence spectrum. Five gold atom sites in the asymmetric unit were found. Several iterations of model building with WinCoot (version 0.8.9)^27^ and restrained refinement with REFMAC5 (ref. ^28^) from the CCP4 package (version 7) and PHENIX program (version 1.17.1)^29^. After repeated model rebuilding and refinement, the final model was refined using SHELXL at 2.15 Å resolution. The structure of OsDAO was determined by molecular replacement with Molrep using the refined structure and OsGA2ox3 as the search model. Model building and refinement were performed as well as OsGA2ox3. The statistics of the data collection and refinement are shown in Supplementary Table 1. Molecular graphics (Figs. 1 and 2, Supplementary Fig. 2) were illustrated with PyMOL^30^ (http://www.pymol.org) and UCSF Chimera^31^ (Fig. 1).

### Gel filtration analysis

Purified monomer OsGA2ox3 and OsGA2ox6 were incubated at 10 mg/ml and 25 °C for 18 h in buffer C (10 mM potassium phosphatase pH 7.4) with 10 mM GA_4_. The incubated sample were fractionated by size exclusion chromatography using a Superdex 200 10/300GL column (GE Healthcare) equilibrated with buffer C and eluted with the same buffer (+1 mM GA_4_) at a flow rate of 0.5 ml/min at 4 °C. Fractions (0.5 ml) were collected, and each peak fraction was analyzed by Blue-Native PAGE. The fractions indicated as in Fig. 2 were used for further experiments. Purified monomer OsDAO was incubated at 10 mg/ml and 4 °C for 18 h in buffer C (10 mM potassium phosphatase pH 7.4) with 10 mM IAA. Size exclusion chromatography was performed as described as for the OsGA2ox3. The fractions indicated as in Fig. 4d were used for further experiments.

### Enzyme assay

The purified OsGA2ox3 and OsDAO were incubated at 30 °C with GA_4_ and IAA (OlChemIm), respectively in 1 ml of 100 mM Tris-HCl (pH 7.9) containing 4 mM ascorbic acid, 4 mM 2-oxoglutaric acid, 0.5 mM FeSO_4_, 4 mM DTT, 2 mg/ml BSA, and 1 mg/ml catalase. The reactions were stopped after 1 h incubation by adding 125 μl of acetic acid. Next, 50 ng of deuterated [^2^H_2_] GA_4_ and IAA (OlChemIm) were used as internal standards for the reactions of OsGA2ox3 and OsDAO, respectively. The solution was passed through a 1 ml C18-SD high-performance extraction cartridge (Empore). After the column was washed with 300 μl of water two times, substances retained on the column were eluted with 150 μl of methanol two times. The methanol eluate was evaporated with dry N_2_ gas. After TMSi (trimethylsilyl) ester–TMSi ether derivatization with MSTFA (*N*-methyl-*N*-trimethylsilyl-trifluoroacetamide), the GA_34_ and oxIAA were analyzed by gas chromatography-selected ion monitoring–mass spectrometry. Full-scan GC-MS analysis of GAs was performed using a mass spectrometer (JMS-K9; JEOL) connected to a gas chromatograph (6890N; Agilent Technology). The trimethylsilylated derivatives (TMSi ester–TMSi ether) were injected (285 °C) into an DB-1 column (0.32 mm i.d. × 30 m, 0.25 μm film thickness; Agilent Technology). The column temperature was kept at 120 °C for 2 min, then increased at a rate of 20 °C/min to 260 °C and held for 1 min, and then increased at a rate of 4 °C /min to 320 °C. The flow rate of the carrier He gas was 1.5 ml/min, and mass spectra were acquired by scanning from *m*/*z* 50 to 750 at 70 eV.

### RNA isolation and quantitative RT-PCR analysis

Total RNAs were purified from young seedlings using RNeasy Plant Mini Kit according to the manufacturer’s guidelines (Qiagen) and treated with RNase-free DNase (Qiagen). Three biological replicates were analyzed using RNAs isolated independently. The first strand of cDNA was synthesized from 1 μg of total RNA using the Omniscript reverse transcription kit (Qiagen). Real- time RT-PCR analysis was performed on a CFX96 real-time PCR detection system (Bio-Rad) with the SYBR Green PCR kit (Qiagen) and appropriate primers. The ubiquitin gene was used as an internal standard for normalizing variations in cDNA concentrations.

### Production of transgenic rice plants and their growth conditions

The OsGA2ox3/pBI, OsGA2ox3/pCAMBIA 1380, pOsGA2ox3-WT/pSTARA, pOsGA2ox3-3Mu/pSTARA, pSTARA vector control, and OsGA2ox3 knockout mutant were transferred into *Agrobacterium tumefaciens* stain EHA105 by electroporation. These EHA101 stains were grown for 3 days on the AB medium containing 50 mg/l hygromycin and 50 mg/l kanamycin or 100 mg/l spectinomycin solidified with 1.5 % agar. The bacterial cells were resuspended in AAM medium. The calli (*Oryza sativa* L. cv. Nipponbare) were soaked in this suspension for 2 min and blotted dry, using sterile Kimwipes for removal of excess bacteria. Then these calli were transferred on a piece of filter paper placed on a co-culture medium. A co-culture medium was prepared by spreading 5.35 ml of a liquid medium (2N6-AS medium)^32,33^ on a bottom medium. After co-cultivation, the calli were washed three times with water containing 0.6 mg/l acetosyringone. After washing, the transgenic calli were selected on a medium containing 25 mg/l meropenem (Wako) and antibiotics corresponding to each constructs. Seedlings were established on Murashige and Skoog (MS) medium [half-strength MS salt mixture (pH 5.7; Wako), B_5_ vitamins, 1% sucrose, and 0.8% gellan gum (Wako)]. Rice plants, expect when used for protoplast isolation, were grown in a greenhouse at 30 °C under a 16 h light (long-day treatment) and 10 h (short-day treatment). Plants (rice T65) used for protoplast isolation were grown in an incubator at 30 °C for 6 days.

### β-Glucuronidase (GUS) staining

The construct of OsGA2ox3::GUS was transformed into *Agrobacterium tumefaciens* stain EHA105. Various organs of transgenic lines were prepared for histochemical GUS staining^34^. Transgenic rice was grown in greenhouse, and before and after heading plants were collected and vacuum infiltrated for 10 min with GUS staining buffer (50 mM sodium phosphate, pH 7.0, 1 mM 5-bromo-4-chloro-3-indolyl-β-d-glucuronide and 7% [v/v] methanol). After incubation in darkness for 18 h at 37 °C, various tissues were completely cleared with 70% ethanol.

### Bimolecular fluorescence complementation assay

Plasmids were extracted using the NucleoBond Xtra Midi Plus according to the manufacturer’s instruction (Takara). Leaves (rice T65) were collected from 6-day-old plants grown. The rice T65 mesophyll protoplasts were performed^35^ and calculated with the modification that the protoplasts were isolated using the Tape-sandwich method^36^. YFP fluorescence was recorded by a LSM 700 confocal microscope (Zeiss).

### Phylogenetic analysis

Alignment analyses on amino acid sequences was accomplished using MAFFT (version 7)^37^ with the L-INS-i model, and then manually adjusted to optimize alignments using SeaView (version 4). Bayesian estimation of phylogenetic topology was conducted with MrBayes (version 3.2.6)^38^, using the General Time Reversible (GTR) + site-specific rates model (SS) model. Four chains of the Markov chain Monte Carlo (MCMC) analyses were run simultaneously and sampled every 1000 generations for a total of 1,100,000 generations.

### MD setup

The force field parameters for α-ketoglutalic acid and GA_4_ were constructed using the generalized amber force field (GAFF)^39^. Charges of atoms were determined from the restrained electrostatic potential (RESP)^40,41^ calculation using Gaussian09 (ref. ^42^). AMBER 14SB force field^43^ has been used to model the protein. A tetramer of OsGA2ox3 residue IDs 12–326 was selected for the simulation, where N- and C-termini of each subunit of OsGA2ox3 were capped by acetyl and methyl groups, respectively. Four cysteines at residue 194 were connected by two S–S bonds. All histidines except His182 were protonated at Nε, while His182 residues were protonated at Nδ. The GA_4_ molecule at the A/D subunit intermolecular site was removed from the initial configuration. The system was then solvated in 150 mM sodium chloride solution with the truncated dodecahedron periodic boundary. The periodic boundary was set to ensure at least 10 Å thickness from the protein. In the end, 59,940 molecules of TIP3P water, 202 sodium ions, and 185 chloride ions were placed. The total number of atoms was 199,630. After the model was constructed, MD simulation was performed. Throughout all simulations, long-range interactions were calculated with the smooth particle mesh Ewald method^44^ with the real-space cut-off length of 10 Å. The system was first equilibrated in NPT (constant-temperature, constant-pressure condition) for 1 ns with heavy atom restrained to the coordinate from the X-ray crystallographic structure, then the system was further equilibrated with 20 ns NPT run without restraints. The temperature and the pressure were set to 300 K and 1 atm, respectively. The timestep was set to 2 fs with bonds between hydrogens and heavy atoms being constrained.

### Free-energy analysis of the GA_4_ loading mechanism

After the equilibration, the GA_4_ inside the subunit “A” was manipulated by the string method^45^ using the center-of-mass (COM) coordinate as collective variables. Each COM of the GA_4_ was restrained to the guiding path coordinate by a harmonic restraint of 2.39 kcal/mol/Å^2^ (1000 kJ/mol/nm^2^). There were 48 points in the guiding path conforming one loop; guiding points numbered “0” and “47” were considered to be connected during the string method phase. The guiding points numbered “0” and “24” were fixed to the COM of A/D interface GA_4_ in the X-ray crystallographic structure, and that at the active site in the subunit “A”, respectively. The guiding points were optimized with the string method to elucidate a “rough” loading path. After the guiding points were optimized, we performed the replica-exchange umbrella sampling method^46,47^. The combination of the string method and the replica-exchange umbrella sampling method have been shown to be more reliable for the simulation of proteins^48^. The simulation used the optimized guiding points as centers of harmonic umbrella potentials with a force constant of 0.48 kcal/mol/Å^2^ (200 kJ/mol/nm^2^). After 2 ns of the equilibration, the production run was performed for 20 ns. We confirmed the convergence by comparing the free-energy landscape at 15 and at 20 ns. Simulations were performed with GROMACS 2016 (ref. ^49^) extended with PLUMED2 (ref. ^50^). The total simulation time for the production run was thus 960 ns. After the simulation, the free-energy landscape (or the potential of mean force with respect to the GA_4_ coordinates) was constructed using the multistate Bennett acceptance ratio method^51^. The loading pathway was then obtained by another round of string method on the obtained 2D free-energy landscape. Energy barrier was simply estimated as the maximum free-energy difference from the interfacial GA_4_ position. The representative structures for the transition states were obtained from the highest energy states along two paths. Structures were extracted using the weighted resampling from the ensemble corresponding to the grid points. For each grid point, 100 structures were randomly resampled with replacement by the reweighting.

### Additional MD simulation to investigate the role of the gate region

The structure of OsGA2ox3 tetramer with all six GA_4_-binding sites occupied was constructed (199,660 atoms). The initial structure was equilibrated as described previously. Eight independent 90 ns production runs were then performed from the same coordinate but with different initial velocities with the following biasing potentials. To accelerate the GA_4_ to exit the active site, we added the following artificial bias potential to the simulations:

$$V_{{\mathrm{bias}}} = k_{{\mathrm{bias}}}\left( {\min \left( {r - r_0\left( t \right),0} \right)} \right)^2,$$

$r_0\left( t \right) = r\left( {t = 0} \right) + ct,$ where *t* is the time from the beginning of the simulation, *r* is the distance between the COM of the active siteGA_4_, and that of the C-α atoms of 61 residues (residue IDs 89–92, 95, 98–105, 110–114, 176–181, 200–210, 242, 243–245, 259–261, 274–278, and 308–321 of subunit A). These residues were selected so that they are within 10 Å from any atoms of GA_4_ in the initial structure. The constants were set to *k*_bias_ = 10 kJ Å^−2^ and *c* = 0.1 Å/ns, respectively. The RMSF (see Supplementary Note 1 for the definition) of all Cα atoms in subunit A was calculated for each 20 ns of the simulation, and averaged over eight independent runs. The time course of RMSD (see Supplemental Note 1) of the gate region from the initial structure was also calculated for subunit A and subunits BCD.

### Reporting summary

Further information on research design is available in the Nature Research Reporting Summary linked to this article.

## Supplementary information

Supplementary Information

Reporting Summary

## Data Availability

The coordinates are deposited into the Protein Data Bank with accession numbers 6KU3 and 6KUN. All simulation starting models and trajectories are available upon request.

## Source Data

Source Data
