# Supplementary material for: A common allosteric mechanism regulates homeostatic inactivation of auxin and gibberellin
Source: Nat Commun. 2020 May 1;11:2143. doi: 10.1038/s41467-020-16068-0 (PMC7195466; doi:10.1038/s41467-020-16068-0)
Supplement: Supplementary file 1 — Supplementary Information [file 41467_2020_16068_MOESM1_ESM.pdf]

Supplementary Information

A common allosteric mechanism regulates homeostatic inactivation of auxin and gibberellin

S. Takehara et al.

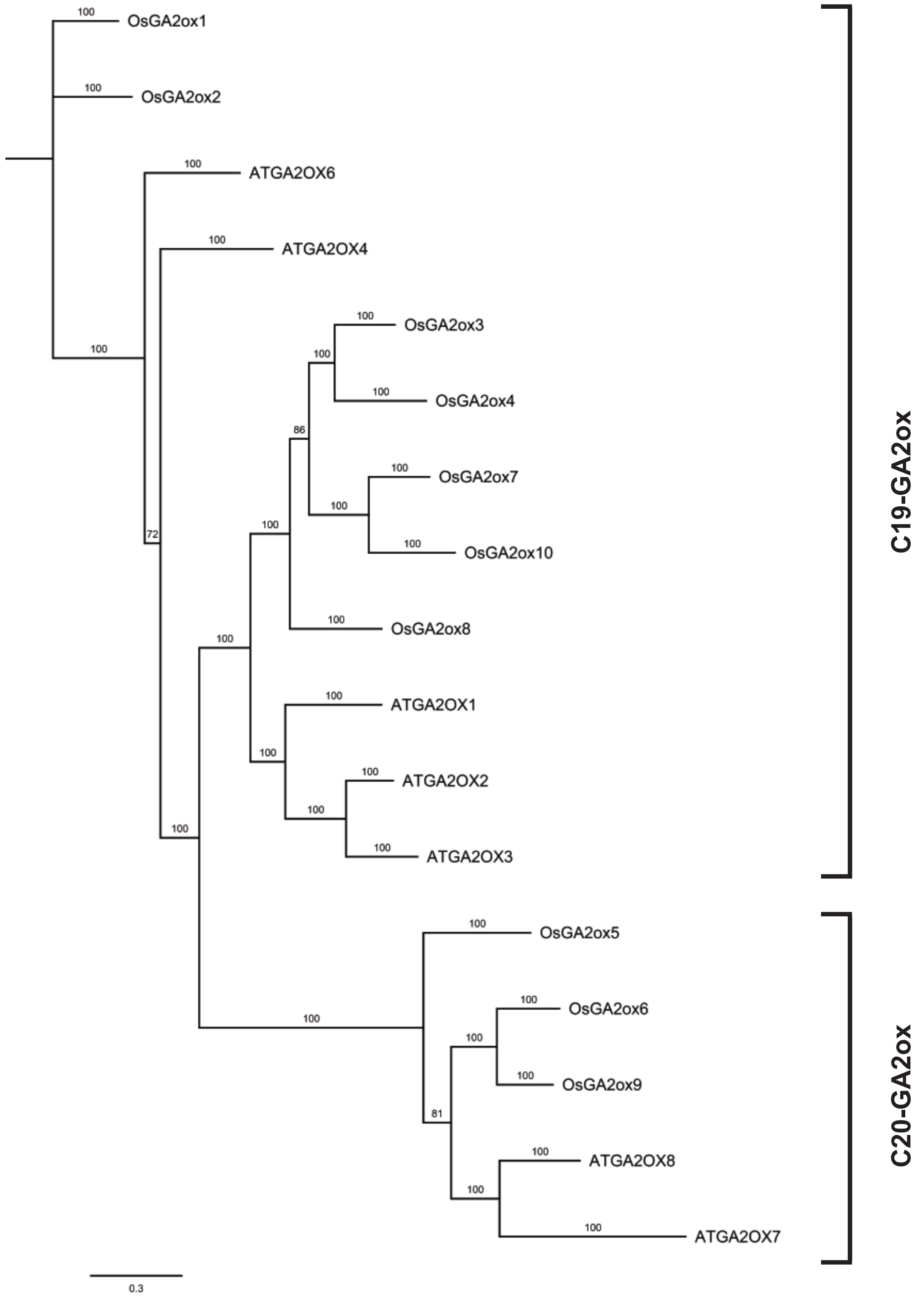

Supplementary Fig. 1: Phylogenetic tree based on the characterization of amino sequences alignment of OsGA2oxs from *O. sativa* and *A. thalliana*.

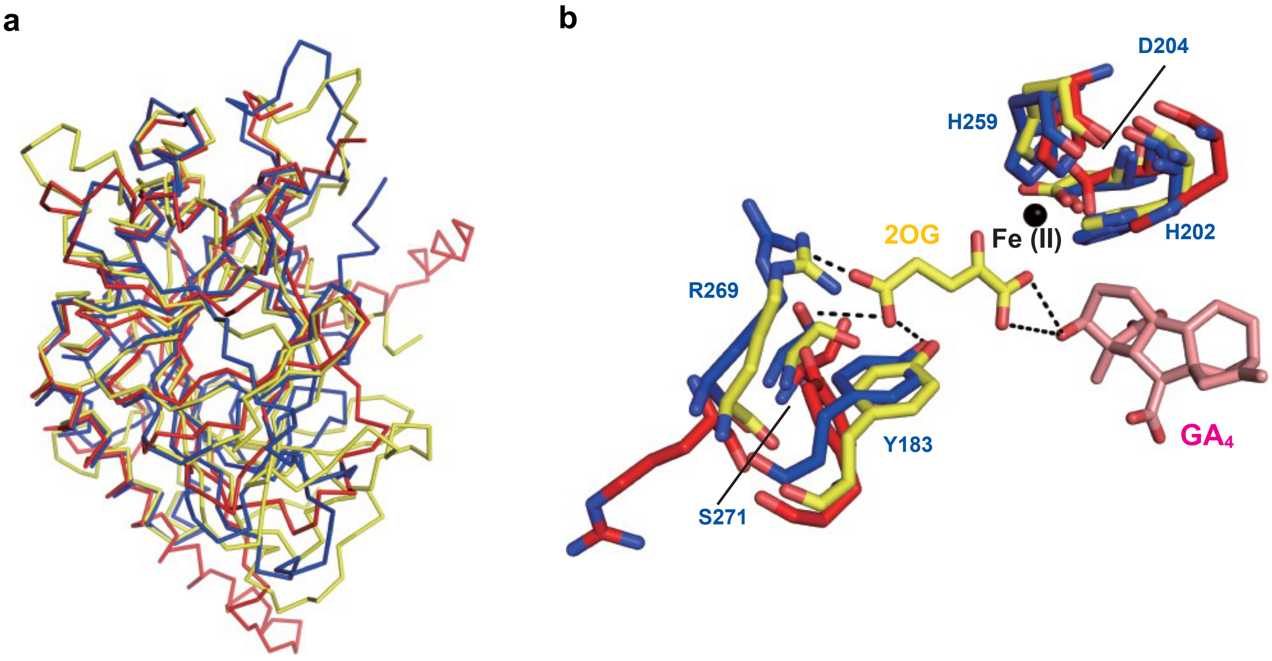

Supplementary Fig. 2: OsGA2ox3 structure superimposed with 2ODDs structure. a, The crystal structure of OsGA2ox3 is represented in blue superimposed against ANS (PDB ID: 1GP5, yellow) and ACCO (PDB ID: 1W9Y, red). Protein backbone is shown in ribbon. **b,** Close-up view of the active site in panel a. GA_4, 2OG and Fe (II) bound to active site are shown in pink, yellow and black, respectively. Protein residues are shown in stick. Hydrogen bonds are indicated by black dashed lines. Amino acids numbered in blue represent those in OsGA2ox3.

Supplementary Fig. 2 S.Takehara et al.

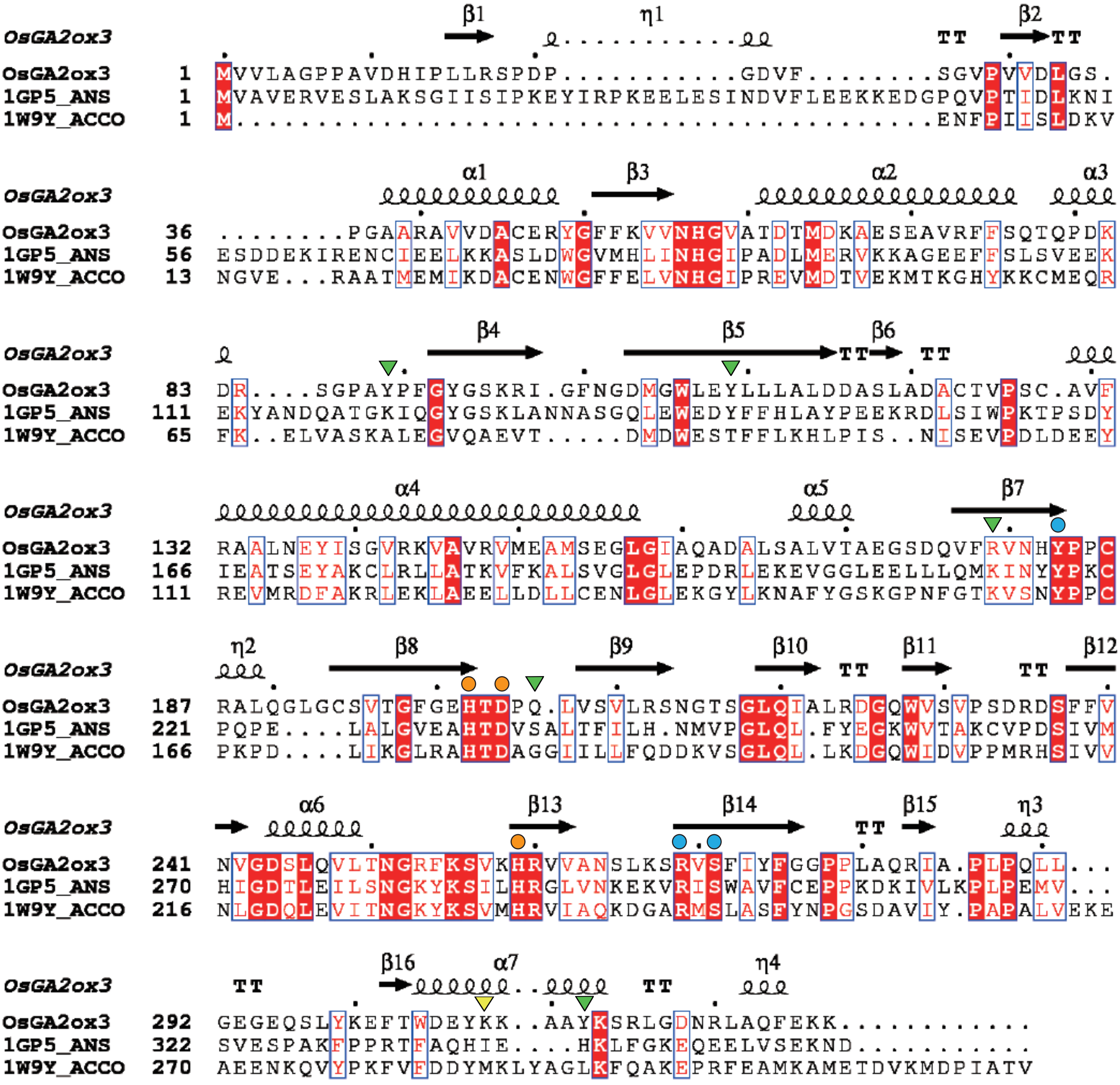

Supplementary Fig. 3: Amino acid sequence alignment of OsGA2ox3 and 2ODD. Indicated sequences of rice GA2ox3, ANS and ACCO were aligned using MAFFT algorithm (https://www.ebi.ac.uk/Tools/msa/mafft/) and the graphic was prepared on the ESPript 3.0 online server (http://espript.ibcp.fr). Labels are as follows: white letters on red background box are for strict identity; red letters indicate similarity in a group and character is illustrated with a blue frame for similarity across groups. The secondary structure elements observed in OsGA2ox3 structure (flat arrows for β-sheets, helices for α- and 3_{10}- (η) helices and TT for β-turns) are shown on the top. The important residues for Fe (II) and 2OG binding are highlighted in orange and blue closed circles above the alignment, respectively. Green and yellow closed triangles indicate the residues binding to GA_4 in the active site and intermolecular GA_4, respectively.

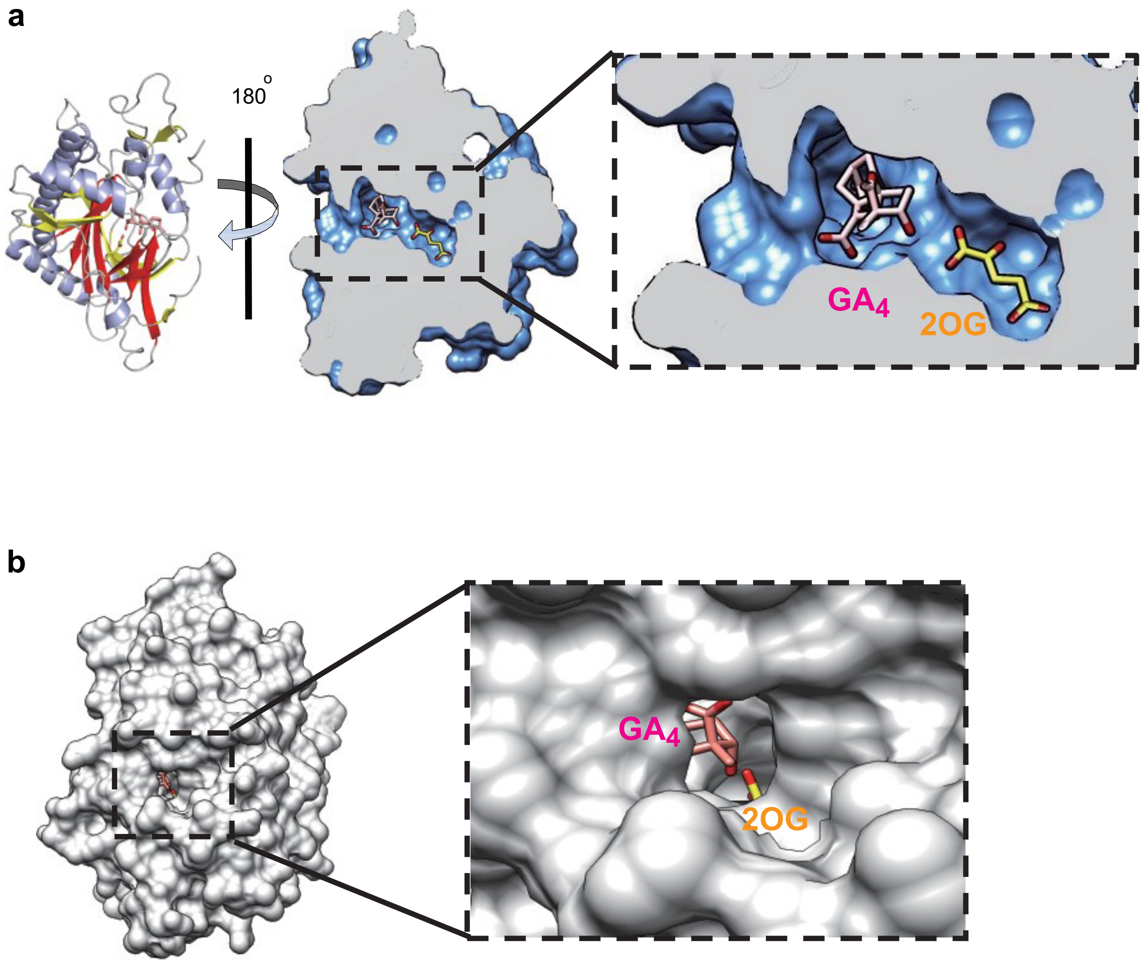

Supplementary Fig. 4: OsGA2ox3 structure of one subunit. a, Cross-sections of the surface area showing a tunnel-like pocket of the active site. Close-up view of cross-section (right panel) rotated 180 clockwise relative to overall view (left panel). **b,** Close-up view of the surface structure. GA_4 and 2OG bound to the active site are shown in pink and yellow stick, respectively.

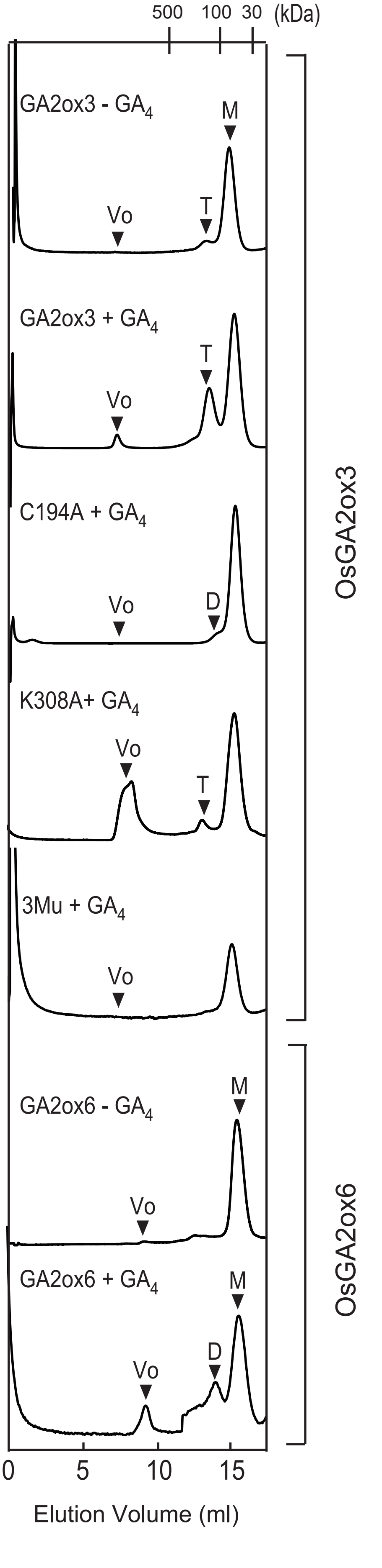

Supplementary Fig. 5: The gel filtration profile of WT-OsGA2ox3, WT-OsGA2ox6, and their mutation derivatives with or without GA_4. 3Mu is OsGA2ox3 carrying C194A, K308A, and K313A. T, D, M and Vo indicate the expected elution positions for tetramers, dimers and monomers of OsGA2oxs and void volume.

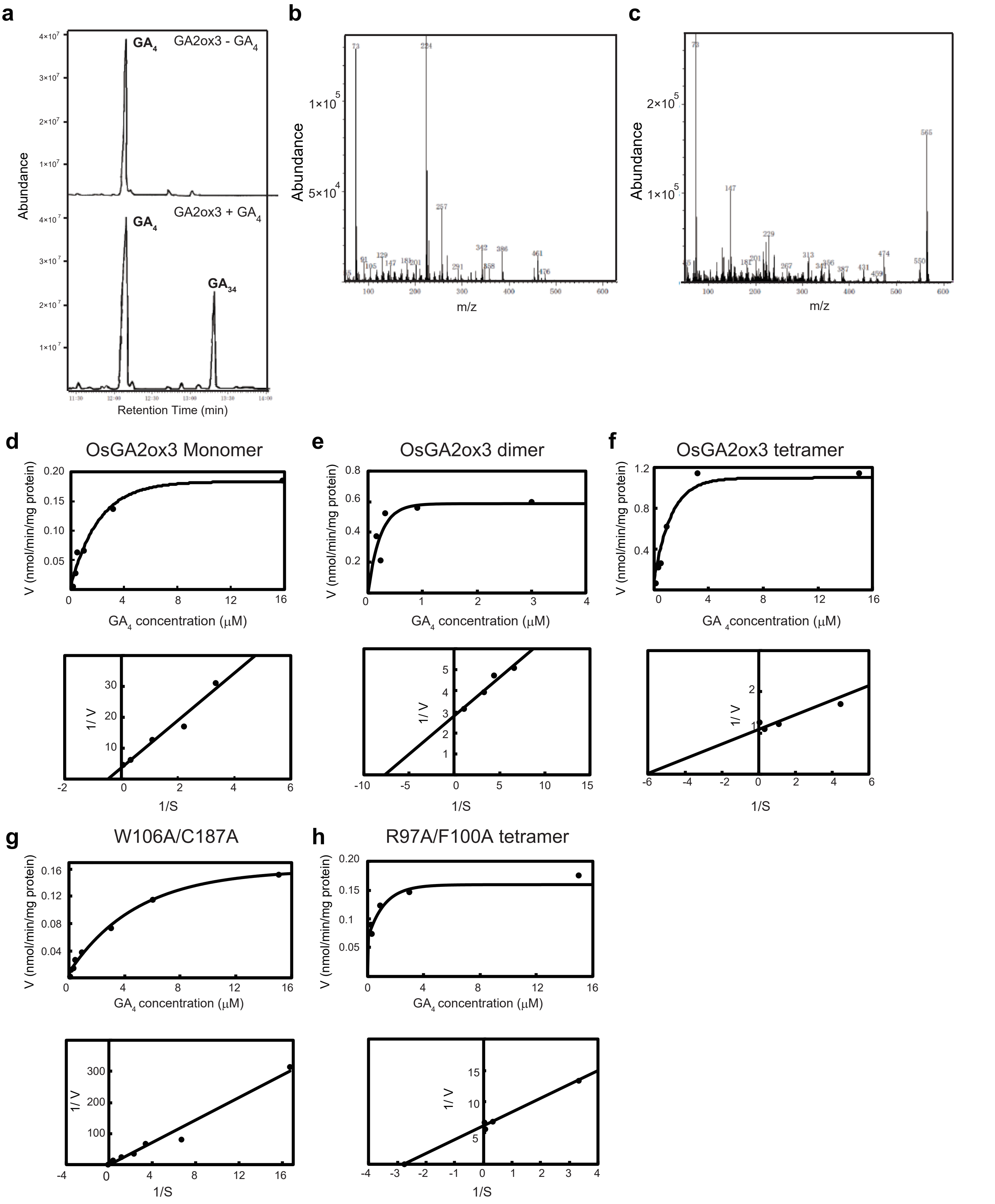

Supplementary Fig. 6: Enzymatic analysis of OsGA2ox3. **a,** Gas chromatogram of the reaction mixture for OsGA2ox3 (bottom panel) or no enzyme (top panel) in the presence of GA_4. The positions of the substrate GA_4 and products GA_{34} are indicated in the panels. **b and c,** Substrate (b) and product (c) were identified by mass spectrometry. Spectra were obtained by daughter ion-scanning negative ions from m/z 50 to 650 with collision energy at 70 eV. **d-f,** Enzymatic analyses of monomer, dime and tetramer structure of OsGA2ox3. **g and h,** Enzymatic analyses of W106A/C187A and R97A/F100A mutant proteins. Michaelis-Menten (top panel) and Lineweaver-Burk (bottom panel) plots for each reaction.

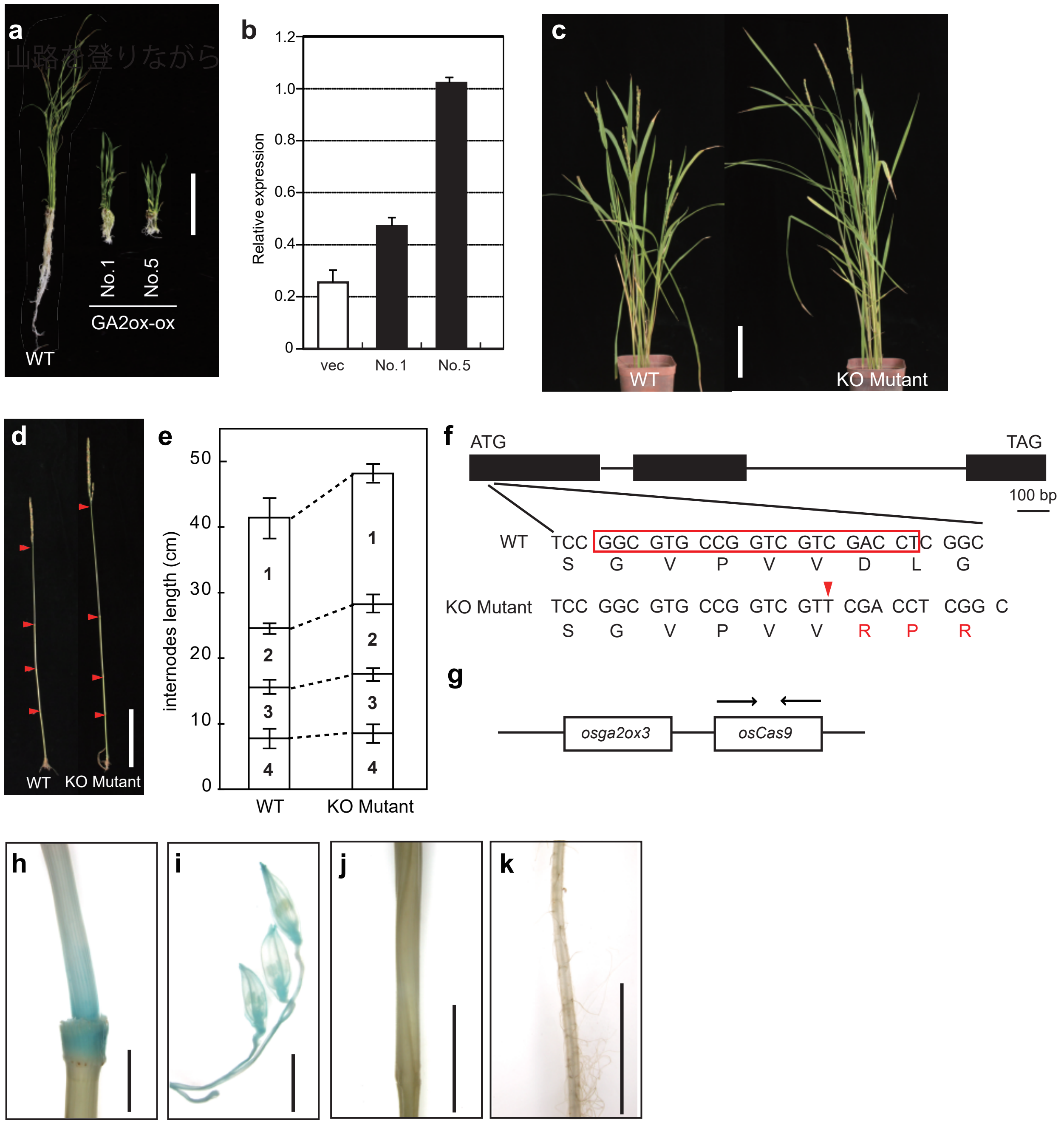

Supplementary Fig. 7: OsGA2ox3 functions in GA inactivation *in planta*. a and b, *OsGA2ox3*-overexpressor showed more severe dwarfism. **a,** Wild-type (WT) and 2 lines of *OsGA2ox3*-overexpressor driven actin promoter (GA2ox-ox, lines 1 and 5) plants. Bar = 3 cm. **b,** The *OsGA2ox3* expression levels of the plants in Supplymentary Fig. 6a. **c-e,** *OsGA2ox3*-knockout (KO) mutant produced by CRISPR/Cas9 system showed elongated leaf and internode phenotype. Whole plant (c), culm (d), and internode length (e) of WT and KO mutant. Red arrowheads indicate nodes. Bar = 10 cm. **e,** Values are means ± SD (n = 8 plants). **f,** CRISPR-Cas9-induced mutation detected by sequencing. The black boxes and lines indicate the exons and intron, respectively. Selected target sequences (20 base pairs) are shown in red frame. The red arrowhead shows the position of the base insertion site. **g,** Genotyping of *Cas9* in T2 generation among OsGA2ox3 KO mutants. Plants that showed the desorption of *Cas9* by the primer pair (arrows) were further used for the complementation experiment in Fig. 2f and g. **h-k,** GUS activity in the divisional zone and node region of uppermost internodes (h) and spikelets (i) at one week before heading. No staining was observed in flag leaf (j) or the root (k). Bar = 1 cm

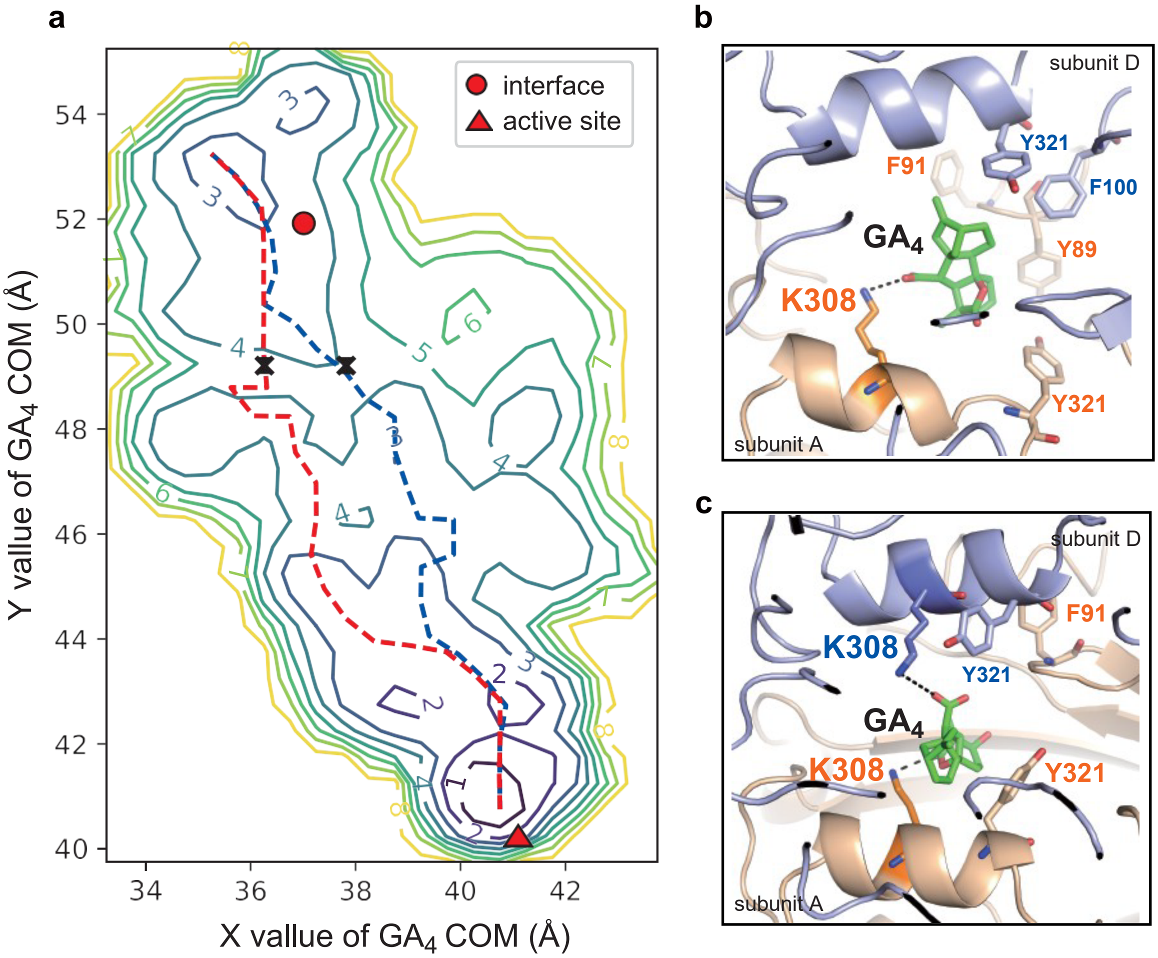

Supplementary Fig. 8: The key interactions to stabilize the intermediate states upon GA_4 loading. a, Same free-energy landscape of the GA_4 loading process as in Fig. 3b. Black crosses represent the transition states used to sample structures. **b,** Representative structure of K308-GA_4 interaction at the transition states. When GA_4 follows the "blue" path, K308 forms a salt bridge to the GA_4 (green stick), and GA_4 is supported by other residues (Y89, F91, F100 and Y321) via hydrophobic interactions. **c,** For the case of "red" path, K308 forms a hydrogen bond to oxygen of γ-lactone ring of GA_4, while K308 of subunit D forms a salt bridge to the C6 carboxyl group. Residues close to GA_4 are also presented in stick form.

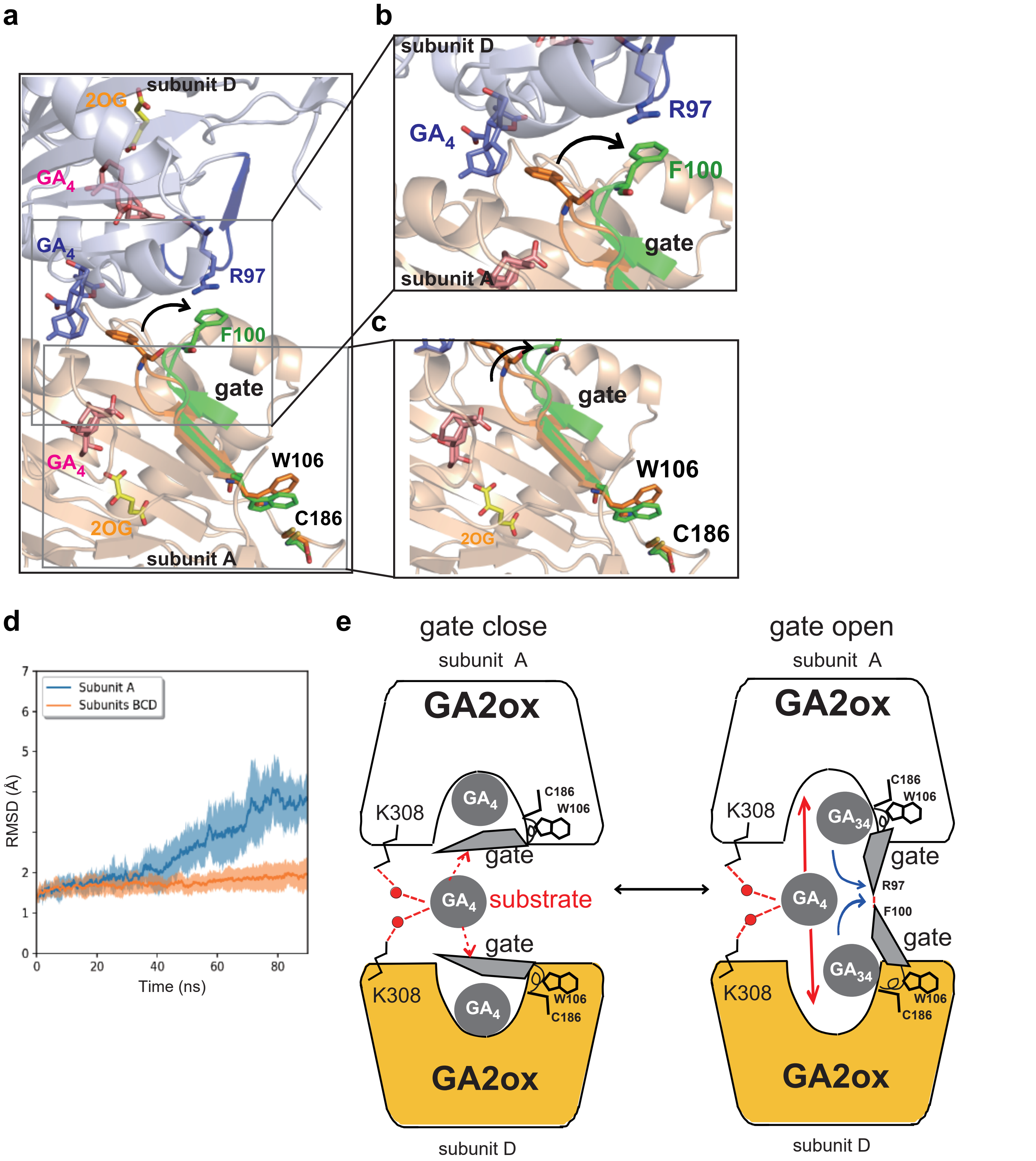

Supplementary Fig. 9: Dimerization of OsGA2ox enhances its enzymatic activity. a, The initial structure of the gate (gate close, Extended Data Fig. 8e) is coloured green, and gate structure at 90 ns of simulation for run 3 is shown in orange (gate open, Extended Data Fig. 8e). The position of the active site and interface GA_4 are also presented in pink and purple, respectively. **b,** F100 of subunit A binds to R97 of subunit D to stabilize the opened gate. The snapshot was taken from a simulation run 1 at 88.5 ns. **c,** W106 and C186 are involved in opening the gate as a hinge. **d,** The structure of the subunit A gate deviates from the initial structure upon GA_4 exit from subunit A, whereas the other subunit gates remain stable. **e,** OsGA2ox3 oxidases bioactive GAs, such as GA_4, and converts them into inactive ones, such as GA_{34}. When GA concentration is limited, OsGA2ox3 acts in a monomer conformation. When GA concentration is higher, OsGA2ox3 becomes a tetramer with the aid of interface GA_4 in addition to a GA_4 in the active site. GA_4 is retained in a stable interface position, allowing two subunits to enter the active site for the next reaction without a high energy barrier. K308 is the most important amino acid for retaining GA_4 and for entering the active site. Furthermore, MD simulation revealed the presence of a gate, allowing substrate to enter the active site and for product to exit. This gate had a hinge site composed of three amino acids, W106, C186, and V196, and was also stabilized by the interaction between R97 in subunit A and F100 in subunit D. GA_4-dependent dimerization enhanced its enzymatic activity (by hyper-activation). These mechanisms are conserved in all rice GA2oxs.

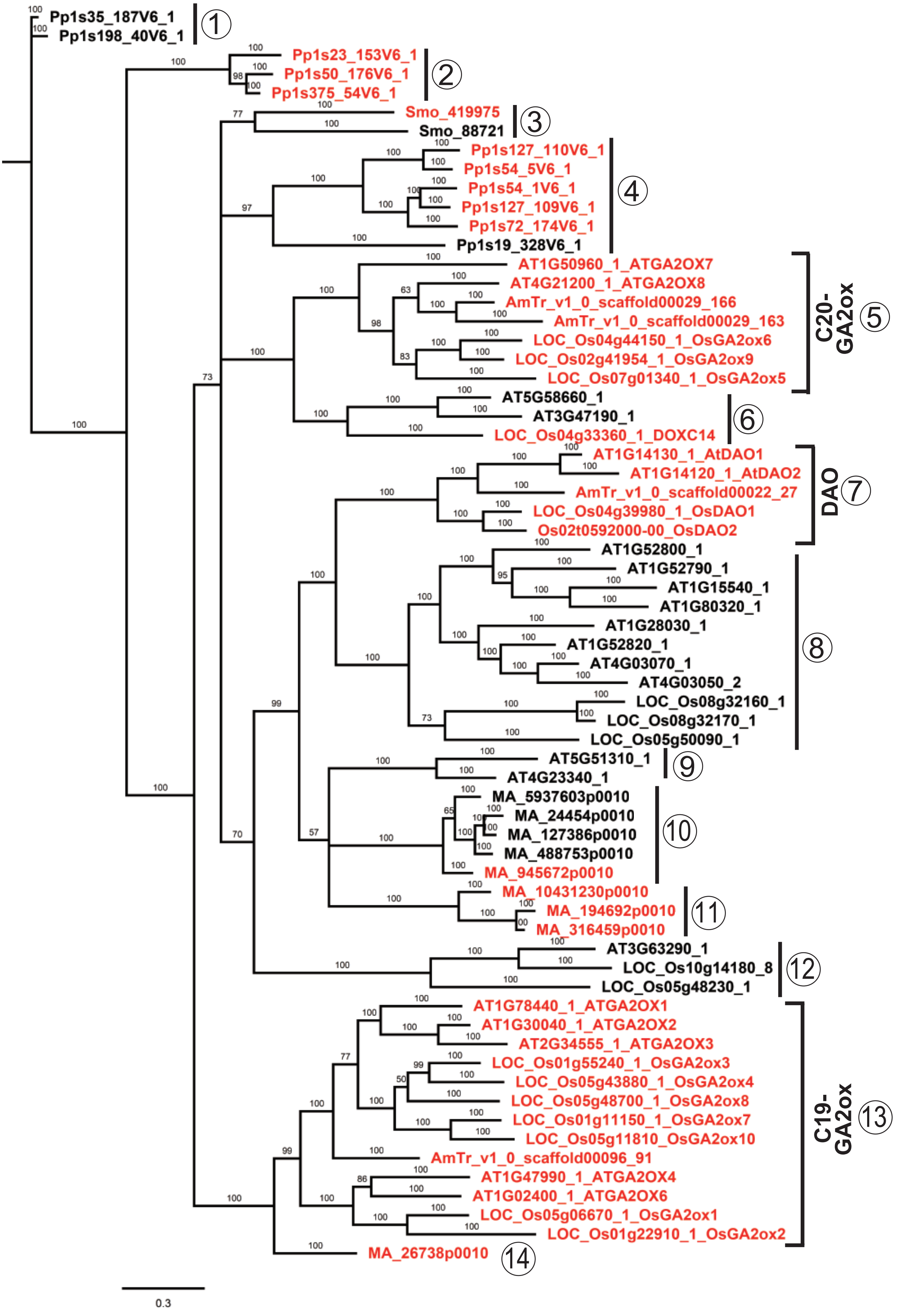

Supplementary Fig. 10: Phylogenetic tree of 2ODD enzymes among *P. patens*, *S. moellendorffii*, *P. abies*, *O. sativa*, and *A. thaliana*. The tree was drawn according to results generated by MAFFT 7.0 analysis using the neighbour-joining method with an amino acid. 2ODD that have Arg or Lys corresponding to the 308th Lys in OsGA2ox3 are shown in red characters in this phylogenetic tree. Each box corresponds to each clade with the same number as in Fig. 5.

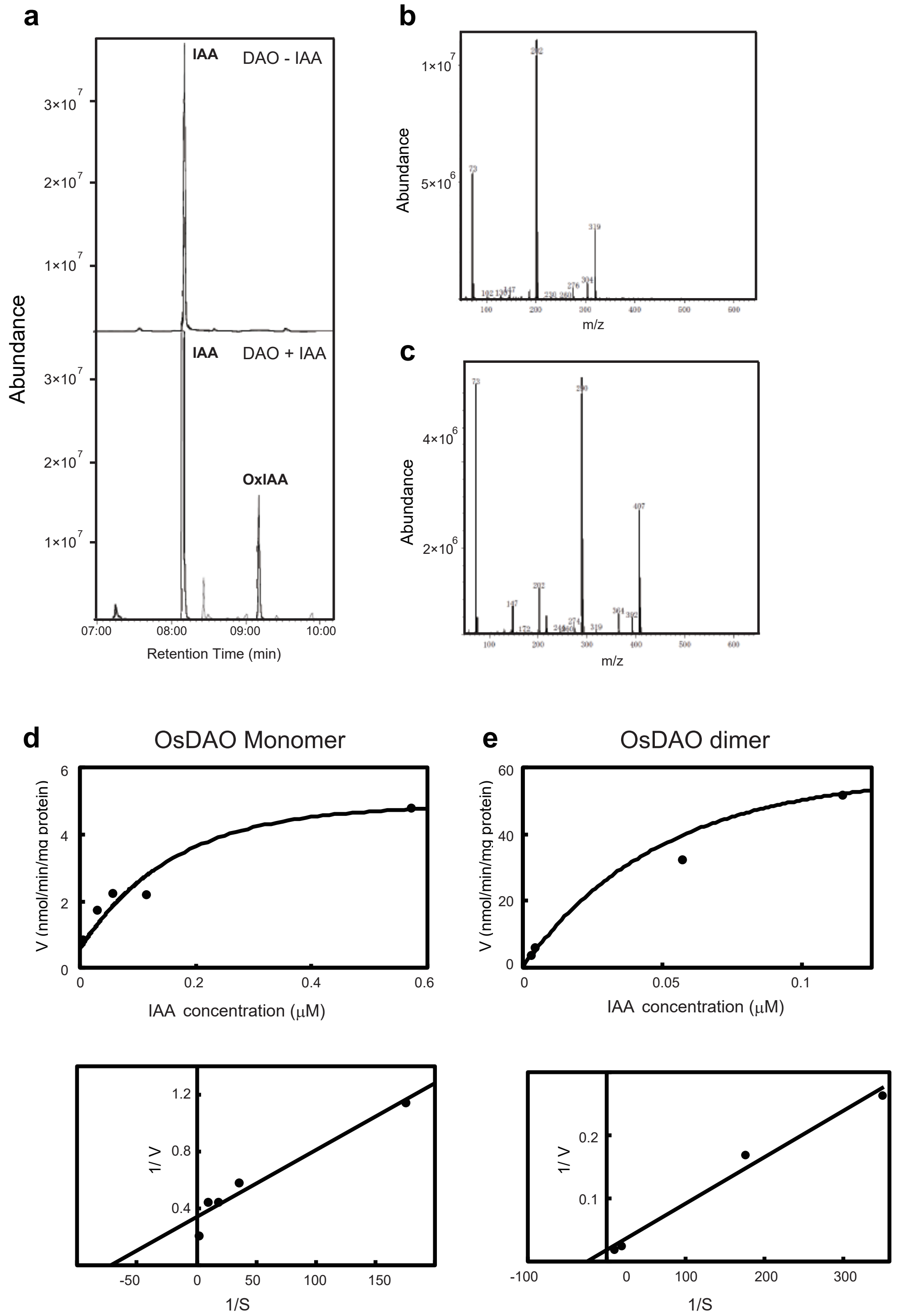

Supplementary Fig. 11: Enzymatic analysis of OsDAO. **a,** Gas chromatogram of the reaction mixture for OsDAO (bottom panel) or no enzyme (top panel) with IAA. The positions of the substrates IAA and products OxIAA are indicated in the panels. **b and c,** Substrate (b) and product (c) were confirmed by mass spectrometry. Spectra were obtained by daughter ion-scanning negative ions from m/z 50 to 650 with collision energy at 70 eV. **d and e,** Enzymatic analyses of monomer and dime structure of OsDAO. Michaelis-Menten (top panel) and Lineweaver-Burk (bottom panel) plots for each reaction.

Supplementary Table 1. Data collection and refinement statistics.

	OsGA2ox3-SAD	OsGA2ox3	OsDAO
**Data collection**			
Beam line	SPring-8 BL26 B1	SPring-8 BL26 B1	SPring-8 BL26 B1
Wavelength (Å)	1.0	1.0	1.0
Detector	MX225HE	MX225HE	MX225HE
Space group	P212121	P212121	P212121
Molecules (Asymmetric Unit)	4	4	2
Cell dimensions			
a, b, c (Å)	98.54, 112.54, 149.28	99.47, 112.74, 149.51	75.25, 78.30, 94.00
α, β, γ (°)	90.00, 90.00, 90.00	90.00, 90.00, 90.00	90.00, 90.00, 90.00
Resolution range (Å)*	50.00-3.10 (3.15-3.10)	50.00-2.15 (2.19-2.15)	50.00-2.0 (2.03-2.00)
Total reflections	205120	456356	362446
Unique reflections	30734	91711	38012
Completeness (%)*	99.4 (99.5)	99.8 (100.0)	99.8 (100.0)
R_{sym} (%)*	6.9 (60.1)	8.2 (60.7)	5.4 (29.0)
$I / \sigma I$*	40.7 (4.7)	22.8 (2.0)	36.2 (6.5)
Redundancy*	6.7 (6.4)	5.0 (4.9)	9.5 (9.6)
**Refinement**			
R_{work} / R_{free} (%)		20.2 / 24.8	20.3 / 25.2
No. of atoms			
Protein		1262	588
Water		402	311
Ligand		274	70
B-factor (Å)			
Protein		44.2	35.7
Water		44.9	34.9
Ligand		51.7	60.2
R.m.s. deviations			
Bond lengths (Å)		0.015	0.012
Bond angles (°)		1.38	1.4
Ramachandran plot			
Favoured (%)		96.01	97.07
Allowed (%)		3.83	2.76
Outlier (%)		0.16	0.17

Highest resolution shell is shown in parenthesis.

Supplementary Table 2. List of primers used in this paper.

No.	Primer name	Sequence (from 5' to 3')
**Enzyme assay & gel filtration analysis**		
1	BamHI_pGEX-OsGA2ox3_Fwd	ccggatccATGGTGGTTCTCG
2	SmaI_pGEX-OsGA2ox3_Rev	cccccgggCTACTTCTTCTC
3	BamHI_pGEX_DAO_Fwd	*ccggatcc*ATGGTGGAGATCCCG
4	NotI_pGEX_DAO_Rev	*ccgcggccgc*TCAGGCCGCCAGAC
5	BamHI_pGEX-OsGA2ox6_Fwd	*ccggatcc*ATGCCGGCCTTC
6	SmaI_pGEX-OsGA2ox6_Rev	*cccccggg*TTATTGTACTGAAG
7	OsGA2ox3_C194A_Fwd	CTCGGCGCGAGCGTCACCGGCTTCGGC
8	OsGA2ox3_C194A_Rev	GACGCTCGCGCCGAGCCCCTGCAGCGC
9	OsGA2ox3_K308A_Fwd	GAGTACGCGAAGGCTGCCTACAAATCA
10	OsGA2ox3_K308A_Rev	AGCCTTCGCGTACTCATCCCATGTGAA
11	GA2ox3_K313A_Fwd	GCCTACGCATCAAGGCTTGGAGACAAC
12	GA2ox3_K313A_Rev	CCTTGATGCGTAGGCAGCCTTCTTGTA
13	GA2ox3_W106A_Fwd	ATGGGGGCGCTCGAGTACCTCCTCCTC
14	GA2ox3_W106A_Rev	CTCGAGCGCCCCCATGTCGCCATTGAA
15	GA2ox3_C187A_Fwd	CCGCCGGCGCGCGCGCTGCAGGGGCTC
16	GA2ox3_C187A_Rev	CGCGCGCGCCGGCGGGTAGTGGTTCAC
17	GA2ox3_F100A_Fwd	ATCGGGGCGAATGGCGACATGGGGTGG
18	GA2ox3_F100A_Rev	GCCATTCGCCCCGATCCGCTTGCTGCC
19	GA2ox3_R97A_Fwd	AGCAAGGCGATCGGGTTCAATGGCGAC
20	GA2ox3_R97A_Rev	CCCGATCGCCTTGCTGCCGTACCCGAA
**BiFC assay**		
21	pENTR_OsGA2ox3_Fwd	*cacc*ATGGTGGTTCTCGCTG
22	pENTR_OsGA2ox3_Rev	CTTCTTCTCAAACTGGGCC
23	pENTR_OsGA2ox6_Fwd	*cacc*ATGCCGGCCTTCGCCGACATC

24	pENTR_OsGA2ox6_Rev	CCTTTTGTACTGAAGAATGC
25	OsGA2ox6_R332A_Fwd	GAGTACGCGAAGAAGGTGCAGGAAGAC
26	OsGA2ox6_R332A_Rev	CTTCTTCGCGTACTCCCCGAAGGTGAA
27	pENTR_ OsDAO_Fwd	*cacc*ATGGTGGAGATCCCGGCG
28	pENTR_ OsDAO_Rev	GGCCGCCAGACGCGCGAGC
**Transgenic plants**		
29	HindIII_pSTARA_GA2ox3_Fwd	*ccaagctt*GCTTTGCCTGC
30	XbaI_ pSTARA _GA2ox3_ Rev	*cctctaga*GGCCAATAGCGTG
31	XbaI_GUS_Hm_GA2ox3_Fwd	*cctctaga*GCAATATACTGTACACACCG
32	SmaI_GUS_Hm_GA2ox3_Rev	*cccccggg*GACGTTGACGAAGAAGGAGTC
33	PCAMBIA_OsGA2ox3_Fwd	*cctctaga*ATGGTGGTTCTCG
34	PCAMBIA_OsGA2ox3_Rev	*cccccggg*CTTCTTCTCAAAC
**Knockout mutant**		
35	SalI_GA2ox3_CRISPR_Fwd	*gttg*GGCGTGCCGGTCGTCGACCT
36	SalI_GA2ox3_CRISPR_Rev	*aaac*AGGTCGACGACCGGCACGCC
37	HincII_GA2ox3_CRISPR_Fwd	*gttg*GCCTAGTGAGCGGGTCAACC
38	HincII_GA2ox3_CRISPR_Rev	*aaac*GGTTGACCCGCTCACTAGGC
39	GA2ox3_SalI_genotype_Fwd	CTCTCCTGCCCTGTTTCTTG
40	GA2ox3_SalI_genotype_Rev	GCGCACCATGTCAAACACTA
41	OsCas9_1394_1413	CCCGCAAGTCTGAAGAAACT
42	OsCas9_2137_2118	ATACCTGGGCCTTTCTGGAT

Supplementary notes

Definition of RMSF and RMSD

The root-mean-square fluctuation (RMSF) is defined as follows. For i-th atom, the RMSF of the atom, namely RMSF_i, is

$$\mathrm{RMSF}_i = \frac{1}{T}\sum_{t=1}^{T} |(\boldsymbol{x}_i(t) - \overline{\boldsymbol{x}}_i)|^2,$$

where t is the index of the snapshots in MD trajectory, T is the total number of snapshots, $\boldsymbol{x}_i$ is the (3-dimensional) coordinate of ith atom, and $\overline{\boldsymbol{x}}_i$ is the average coordinate of $\boldsymbol{x}_i$ among all snapshots of the simulation. Since molecules in the MD simulation may rotate or translate during the simulation time, we aligned each snapshot to the reference structure (X-ray crystallographic structure) before calculating the RMSF values.

The root-mean-square deviation (RMSD) of a snapshot is defined for a group of N atoms as,

$$\mathrm{RMSD} = \frac{1}{N}\sum_{i=1}^{N} \left|\left(\boldsymbol{x}_i - \boldsymbol{x}_i^{\mathrm{ref}}\right)\right|^2,$$

where $\boldsymbol{x}_i$ is ith atom coordinate of the group, and $\boldsymbol{x}_i^{\mathrm{ref}}$ is that in a reference structure. Similar to the RMSF's case, the structure may rotate or translate during the simulation. Specifically, in this work, we aligned the whole protein structure of a subunit (e.g. subunit A) into that of the X-ray crystallographic structure, then calculated the deviation of gate region of the same subunit (e.g. the gate of subunit A) from the reference structure.
